# Impaired Neuregulin 1 Processing is Associated with Synaptic and Behavioral Abnormalities in a Prenatal Valproic Acid Model of Autism

**DOI:** 10.7150/ijbs.133137

**Published:** 2026-05-18

**Authors:** Yu-Jin Kim, Han-Byeol Kim, Hyo-Min Lim, Yoori Choi, Ran-Sook Woo

**Affiliations:** 1 Department of Anatomy and Neuroscience, College of Medicine, Eulji University, Daejeon, 34824, Republic of Korea.; 2 Department of Nuclear Medicine, Seoul National University Hospital, Seoul, 03080, Republic of Korea.

**Keywords:** autism spectrum disorder (ASD), valproic acid (VPA), neuregulin 1 (NRG1), social deficits

## Abstract

Autism spectrum disorder (ASD) is characterized by deficits in social communication and restricted/repetitive behaviors, yet the molecular mechanisms by which prenatal environmental insults lead to circuit dysfunction remain incompletely understood. Neuregulin 1 (NRG1)-ErbB4 signaling is a key regulator of synaptic development and excitation-inhibition (E/I) balance, but whether altered NRG1 processing contributes to ASD-related phenotypes remains unclear. Here, using a prenatal valproic acid (VPA) rat model, we examined the relationship between NRG1 processing, synaptic integrity, and behavioral outcomes. Prenatal VPA exposure reduced cleaved NRG1 protein without altering *Nrg1* transcript levels and was accompanied by decreased expression of the NRG1 sheddases ADAM10, ADAM17, and BACE1. These alterations were accompanied by attenuated ErbB4-AKT/ERK1/2 signaling, reduced synaptic scaffolding proteins, and impaired dendritic spine maturation in the hippocampus. Behaviorally, VPA-exposed offspring exhibited abnormalities across multiple ASD-relevant domains. Recombinant NRG1β1 administration during adolescence improved repetitive behaviors in both sexes, whereas deficits and rescue effects in social and sensorimotor domains were primarily observed in males. As robust social deficits were not evident in females, subsequent molecular and synaptic analyses were conducted in male hippocampus, where NRG1 restored ErbB4 signaling, synaptic organization, and spine maturity without affecting locomotor activity. Collectively, these findings indicate that altered NRG1 processing is associated with synaptic and behavioral abnormalities in the VPA model. Enhancement of NRG1-ErbB4 signaling modulates these phenotypes, supporting a functionally relevant role of this pathway in ASD-related neurodevelopmental alterations.

## Introduction

Autism spectrum disorder (ASD) is a complex neurodevelopmental condition characterized by deficits in social communication and the presence of restricted/repetitive behaviors 1,2. Despite extensive research, the molecular mechanisms linking prenatal environmental insults to circuit-level dysfunction in ASD remain incompletely understood. Both genetic susceptibility and environmental risk factors contribute to ASD pathogenesis, with prenatal exposure to valproic acid (VPA) representing one of the most widely used environmental models that recapitulate core behavioral and neurobiological features of the disorder 3,4.

Among the molecular pathways implicated in neurodevelopmental disorders, neuregulin 1 (NRG1)-ErbB signaling has been extensively studied due to its critical role in synapse development, interneuron function, and the regulation of excitation-inhibition (E/I) balance 5-7. NRG1 is synthesized as a transmembrane precursor and undergoes regulated processing by enzymes such as ADAM10, ADAM17, and BACE1 to generate biologically active fragments capable of activating ErbB receptors 8,9. In particular, ErbB4 is highly enriched in GABAergic interneurons, including parvalbumin-positive (PV⁺) populations, where it modulates synaptic transmission and network activity 10-12.

Disruption of NRG1-ErbB4 signaling has been associated with multiple neuropsychiatric conditions, including schizophrenia and ASD 13,14. Previous studies have demonstrated that perturbation of this pathway can alter synaptic development, inhibitory circuit maturation, and network synchronization 15,16. However, it remains unclear whether alterations in endogenous NRG1 processing contribute to ASD-related phenotypes, particularly in environmentally induced models.

The hippocampus plays a central role in social memory, sensory integration, and experience-dependent plasticity, and has been implicated in ASD-related behavioral alterations 17,18. In addition, NRG1-ErbB4 signaling is prominently expressed in hippocampal circuits, where it regulates synaptic organization and interneuron-mediated inhibitory control 15,19. These features make the hippocampus a relevant region for examining the relationship between molecular signaling and behavioral outcomes.

In the present study, we used a prenatal VPA rat model to examine whether alterations in NRG1 processing are associated with synaptic and behavioral abnormalities. We assessed multiple behavioral domains relevant to ASD, including repetitive/perseverative behaviors, impulsivity-related responses, sociability, social novelty, social-olfactory learning, and sensorimotor gating. We further evaluated hippocampal NRG1 processing, downstream ErbB4-AKT/ERK1/2 signaling, synaptic protein expression, and dendritic spine morphology.

Finally, we investigated whether systemic administration of recombinant NRG1β1 during adolescence could modulate these molecular and behavioral alterations. By integrating behavioral, biochemical, and structural analyses, this study aims to provide a comprehensive framework linking NRG1-ErbB4 signaling to circuit-level dysfunction in an ASD-relevant model.

## Materials and Methods

### Reagents and antibodies

Recombinant NRG1β1 (CYT-407) was purchased from ProSpec (East Brunswick, NJ, USA). VPA (P4543) was obtained from Sigma-Aldrich (St. Louis, MO, USA).

Primary antibodies were obtained from the following sources: Synapsin1 (AB1543P, rabbit polyclonal, RRID: AB_90757) and BACE1 (MAB5308, mouse monoclonal, RRID: AB_11212616) from Millipore (Chemicon, MA, USA); phospho-AKT (4051S, mouse monoclonal, RRID: AB_331158), AKT (9272S, rabbit polyclonal, RRID: AB_329827), phospho-ERK1/2 (p-p44/42 MAPK; 9101S, rabbit polyclonal, RRID: AB_331646), and ERK1/2 (p44/42 MAPK; 4695S, rabbit monoclonal, RRID: AB_390779) from Cell Signaling Technology (Danvers, MA, USA); NRG1 (sc-393006, mouse monoclonal, RRID: AB_2927545; sc-28916, rabbit polyclonal, RRID: AB_2154793), phospho-ErbB4 (sc-33040, rabbit polyclonal, RRID: AB_2099887), ErbB4 (sc-283, rabbit polyclonal, RRID: AB_2231308), and β-actin (sc-47778, mouse monoclonal, RRID: AB_626632) from Santa Cruz Biotechnology (Santa Cruz, CA, USA); PSD95 (MA1-046, mouse monoclonal, RRID: AB_2092361) from Invitrogen (Thermo Fisher Scientific, Waltham, MA, USA); and ADAM10 (ab1997, rabbit polyclonal, RRID: AB_302747) and ADAM17 (ab39162, rabbit polyclonal, RRID: AB_722565) from Abcam (Cambridge, UK).

### Animals and VPA-induced autism-like models

Sprague-Dawley (SD) rats were obtained from Samtako Bio Korea (Osan, Republic of Korea) and housed under standard laboratory conditions (12-h light/dark cycle, 23 ± 2 °C, 40-60% humidity) with ad libitum access to food and water. All experimental procedures were approved by the Institutional Animal Care and Use Committee of Eulji University (EUIACUC 22-21).

Female rats were paired overnight, and the presence of a vaginal plug the following morning was designated as embryonic day 0.5 (E0.5). On embryonic day 12.5 (E12.5), dams in the VPA group received a single intraperitoneal (i.p.) injection of VPA (500 mg kg⁻¹; Sigma-Aldrich), whereas control dams received an equivalent volume of saline.

Prenatal exposure to VPA is a well-established environmental model of ASD that recapitulates core behavioral and neurobiological features of ASD 3,4. A total of 70 dams were used, and pregnant dams were randomly assigned to either the SAL or VPA group. Offspring from multiple litters were used for each experimental group: (i) General behavioral tracking was performed in both male (SAL *n* = 60, VPA *n* = 65) and female (SAL *n* = 40, VPA *n* = 40) offspring, (ii) For the STFP test, a separate cohort was used, including males (SAL* n* = 23, VPA *n* = 20) and females (SAL *n* = 17, VPA *n* = 15), with no animals excluded from the analysis, and (iii) For the PPI test, an independent cohort was used, including males (SAL *n* = 17, VPA *n* = 17) and females (SAL *n* = 13, VPA *n* = 14).

To minimize potential litter effects, offspring from different litters were distributed across experimental groups. Animals that did not meet predefined inclusion criteria or were lost during the experimental period (e.g., mortality or technical exclusion) were excluded from analysis. The individual animal was treated as the unit of analysis.

Pups were weaned at postnatal day (PND) 21 and subsequently group-housed (2-3 animals per cage) to minimize stress associated with social isolation. In (ii) and (iii) behavioral testing, animals were single-housed only during the habituation period. Behavioral testing was conducted between PND32 and PND45. All other behavioral assays were performed in the same animals in a fixed sequence.

Recombinant NRG1β1 (50 pg g⁻¹, i.p. injection) was administered once daily for 5-10 consecutive days prior to behavioral testing. The exact number of injections varied depending on the experimental schedule and behavioral paradigm. This dose was selected based on previous studies demonstrating that low-dose systemic NRG1 is sufficient to activate central ErbB4 signaling without inducing nonspecific effects 20-22.

### Western blot

Western blotting was performed as previously described 23 with minor modifications. Briefly, hippocampal tissues were homogenized in ice-cold lysis buffer containing 20 mM Tris-HCl (pH 8.0), 137 mM NaCl, 1% NP-40, 10% glycerol, 1 mM NaF, 1 mM Na₃VO₄ (phosphatase inhibitors), and protease inhibitors (1 μg/mL each of aprotinin, leupeptin, and pepstatin). Lysates were clarified by centrifugation, and protein concentrations were determined using the Bradford assay. Equal amounts of protein were separated by SDS-PAGE and transferred to PVDF membranes. Membranes were blocked for 1 h at room temperature (RT) in Tris-buffered saline containing 0.1% Tween-20 (TBS-T) supplemented with 5% non-fat dry milk. For detection of phosphorylated proteins, membranes were blocked in TBS-T containing 1% bovine serum albumin (BSA) instead of non-fat dry milk. Membranes were incubated overnight at 4 °C with primary antibodies (1:1000 for NRG1, ADAM10, ADAM17, BACE1, ErbB4, AKT, phospho-AKT, ERK1/2, phospho-ERK1/2, PSD95, Synapsin1, and β-actin; 1:500 for phospho-ErbB4), followed by incubation with horseradish peroxidase-conjugated secondary antibodies (1:5000) for 1 h at RT. Immunoreactive bands were visualized using enhanced chemiluminescence (ECL; Amersham Pharmacia) and detected with a ChemiDoc™ Touch Imaging System (Bio-Rad, Hercules, CA, USA).

### RNA-seq analysis

Total RNA was isolated using TRIzol reagent (Invitrogen) according to the manufacturer's instructions. RNA quality was assessed using an Agilent 2100 Bioanalyzer, and RNA concentration was measured using a NanoDrop spectrophotometer.

For library preparation, 500 ng of total RNA was used with the QuantSeq 3′ mRNA-Seq Library Prep Kit (Lexogen). First-strand cDNA synthesis was performed using oligo(dT) primers, followed by second-strand synthesis using random primers. Libraries were purified, amplified, and sequenced on an Illumina NextSeq 500 platform (single-end, 75 bp reads).

Sequencing reads were aligned to the reference genome using standard alignment software, and gene expression levels were quantified and normalized. Differential gene expression analysis was performed using appropriate statistical methods, and significantly regulated genes were identified based on predefined criteria (e.g., adjusted* p*-value < 0.05).

### Quantitative real-time PCR (qPCR)

Total RNA was extracted from hippocampal tissue using TRIzol reagent (Invitrogen) according to the manufacturer's instructions. Complementary DNA (cDNA) was synthesized from 1 μg of total RNA using a reverse transcription kit (Bio-Rad).

qPCR was performed using SYBR Green chemistry on a LightCycler® 96 system (Roche). The cycling conditions were as follows: initial denaturation at 95 °C for 10 s, followed by 40 cycles of 95 °C for 10 s, 60 °C for 10 s, and 72 °C for 30 s. Gene expression levels were normalized to GAPDH and calculated using the 2^-ΔΔCt method.

All primers were commercially synthesized (Bioneer, Daejeon, Republic of Korea) as follows:NRG1 (F: GTGCAGCCCCATCTCTTGAT, R: AATACCCACTTCAGGCCAGC); ADAM10 (F: AGTCAAGGACCCTGCTGTAC, R: GATCAGATGCTGGGCAAAGG); ADAM17 (F: CGGGGACTTCAGCCTAGCTC, R: CCAGGACGAAAGGCACCAA); BACE1 (F: CCAACCTTCGTTTGCCCAAG, R: GTGCCTGTGGATGACTGTGA); GAPDH (F: TGTGAGGGAGATGCTCAGTG, R: GTGGACCTCATGGCCTACAT).

qPCR analysis was performed to validate transcript-level changes identified in the RNA-seq dataset.

### Immunohistochemistry analyses

Animals were deeply anesthetized and transcardially perfused with saline. Brains were removed and post-fixed in 4% paraformaldehyde (PFA) at 4 °C for 24 h, followed by cryoprotection in 30% sucrose for 3 days. Coronal brain sections (30 μm thickness) were prepared using a cryostat for subsequent analyses. For antigen retrieval, sections were incubated in 0.1 M citrate buffer (pH 6.0) at 95-100 °C for 9 min, allowed to cool for 15 min, and rinsed in cold phosphate-buffered saline (PBS). Endogenous peroxidase activity was quenched by incubation in 3% hydrogen peroxide in PBS for 10 min. After washing in PBS, sections were blocked for 1 h at RT in blocking solution containing 5% normal serum in PBS. Sections were then incubated overnight at 4 °C with primary antibody against pErbB4 (1:200 dilution), followed by incubation for 2 h at RT with biotinylated secondary antibodies.

Subsequently, sections were treated with an avidin-biotin-peroxidase complex (Vectastain Elite ABC kit, Vector Laboratories) for 2 h at RT. Immunoreactivity was visualized using 3,3′-diaminobenzidine (DAB) for 20 min. Sections were mounted with Permount (Vector Laboratories) and imaged using a light microscope (Carl Zeiss AG, Oberkochen, Germany).

### Immunofluorescence analyses

Tissue was rinsed in PBS and then permeabilized with 0.1% Triton X-100 in cold methanol for 10 min at -20 °C. After three additional washes in PBS, the tissues were blocked for 1 h in blocking solution (5% goat serum in PBS) and incubated with primary antibodies overnight at 4 °C (1:100 anti-PSD95, 1:200 anti-Synapsin1). Sections were then incubated with Alexa Fluor® 488- or 594-conjugated secondary antibodies (1:400) for 1 h at RT. Nuclei were counterstained with DAPI (10 μM in PBS) for 5 min. Finally, stained sections were mounted in VECTASHIELD (Vector Laboratories) and imaged with an LSM 880 with Airyscan confocal system (Carl Zeiss AG, Oberkochen, Germany). For quantitative analysis, 2 sections per animal and 2-3 fields per section were analyzed. Regions of interest (ROIs) were defined based on anatomical landmarks, and image analysis was performed blinded to experimental group.

### Golgi staining and dendrite spine analyses

Golgi-Cox staining was performed as previously described 22 using the FD Rapid Golgi Stain™ Kit (FD Neuro Technologies, Columbia, MD, USA) following the manufacturer's instructions with minor modifications for dendritic spine analysis. All procedures were conducted under dark conditions. Brain tissues were immersed in an equal mixture of kit Solutions A and B and incubated at RT for two weeks, with replacement of the solution on the following day. Tissues were then transferred to Solution C and maintained at RT for at least 3 days. Sections were cut at 100 µm thickness using a cryostat microtome at -22 °C, mounted on 3% gelatin-coated slides with Solution C, and air-dried at RT. Slides were stained with a mixture of 1 part Solution D, 1 part Solution E, and 2 parts distilled water for 10 min, rinsed twice in distilled water (4 min each), dehydrated, cleared in xylene, and cover slipped. Sections were then imaged using an LSM 880 with Airyscan confocal system (Carl Zeiss AG, Oberkochen, Germany). Dendritic spines were classified based on established morphological criteria 24-26. Spines with a large bulbous head and narrow neck (mushroom type) were classified as mature, while thin and stubby spines were categorized as immature. The proportion of mature spines relative to total spine number was calculated for each dendritic segment.

### Social three-chamber test

Social behavior was assessed using a three-chamber apparatus (90 × 60 × 30 cm; clear polycarbonate; Panlab SMART® tracking system, Harvard Apparatus, Barcelona, Spain). The arena was divided into three chambers connected by openings (10 cm wide × 5 cm high) allowing free access between compartments. For detailed analysis, an interaction zone was defined as an area extending 10 cm from each social chamber. To evaluate sociability, subject rats were first placed in the central chamber and allowed to freely explore the arena for 10 min for habituation. Following habituation, a novel rat (stranger) was placed in a wire cage in the right chamber, while an identical empty cage was placed in the left chamber. The subject rat was then returned to the center chamber and allowed to explore all three chambers for 10 min.

To evaluate social-novelty-recognition (social novelty), the familiar rat from the sociability test was placed in one side chamber (familiar zone), and a novel rat was placed in the opposite chamber (stranger zone). The subject rat was again introduced into the center chamber and allowed to explore for 10 min. Time spent in each chamber and within the interaction zones was recorded. Animals received recombinant NRG1β1 (50 pg g⁻¹, i.p.) once daily for 5 consecutive days. The apparatus was thoroughly cleaned with 70% ethanol between sessions.

### Open field test (OFT)

The OFT was performed as previously described 27, with minor modifications. OFT was conducted in a square arena (60 × 60 × 30 cm for rats), which was divided into 16 equal squares. The four central squares were defined as the inner zone, the four corner squares as the edge zone, and the remaining peripheral squares as the outer zone. Adolescent rats were allowed to freely explore the arena for 5 min. Distance traveled in the inner, edge, and outer zones, as well as the time spent in each zone and locomotor velocity, were recorded. In addition, the durations of wall-leaning and grooming behaviors were analyzed. Animals received recombinant NRG1β1 (50 pg g⁻¹, i.p.) once daily for 6 consecutive days. The apparatus was thoroughly cleaned with 70% ethanol between sessions.

### Cliff avoidance test (CAT)

The CAT test was performed as previously described 28 to assess impulsive-like behavior in rats. Animals were placed individually in the center of a circular maze (15 cm in diameter, 25 cm in height), and their behavior was observed for 1 h. Jumping latency was analyzed from video recordings. Animals received recombinant NRG1β1 (50 pg g⁻¹, i.p.) once daily for 7 consecutive days. Between trials, the apparatus was cleaned with 70 % ethanol to eliminate residual odors.

### Visual cliff avoidance test (vCAT)

The vCAT was performed as previously described 28 and similar to the CAT, was used to assess impulsive-like behavior. Each rat was placed on the central platform of an acrylic box (60 x 60 x 40 cm) positioned at the edge of a table 70 cm above the floor, and behavior was recorded for 6 min. Jumping latency was analyzed from video recordings as an index of impulsive-like behavior. Animals received recombinant NRG1β1 (50 pg g⁻¹, i.p.) once daily for 8 consecutive days. Between trials, the apparatus was cleaned with 70 % ethanol to remove residual odors.

### Elevated plus maze (EPM) test

The EPM test was used to assess impulsive-like behavior in rats. The plus-shaped apparatus consisted of four arms (two open and two closed) elevated 72 cm above the floor, with each arm measuring 10 cm in width and 100 cm in length. Rats were placed in the center of the maze and allowed to explore for 10 min. Locomotor activity was automatically tracked, and anxiety-like behavior was quantified from video recordings, including the time spent in open and closed-arms, number of arm entries, and distance traveled. Animals received recombinant NRG1β1 (50 pg g⁻¹, i.p.) once daily for 8 consecutive days. The apparatus was thoroughly cleaned with 70% ethanol between sessions.

### Marble burying test (MBT)

The MBT was conducted in a home cage (43 x 23 x 20 cm) containing a 5 cm layer of corncob bedding. Twenty-five glass marbles (14 mm in diameter) were evenly arranged in a 5 x 5 grid on top of the bedding. Each rat was placed in one corner of the cage, and behavior was recorded. Digital images of the marbles were captured at the end of the session. After 30 min, the numbers of buried and displaced marbles were quantified from the images. A marble was considered buried when more than two-thirds of its surface was covered by bedding 29,30. Animals received recombinant NRG1β1 (50 pg g⁻¹, i.p.) once daily for 9 consecutive days.

### Nest shredded test (NST)

The NST was conducted in a home cage (43 × 23 × 20 cm) containing a 5-cm layer of corncob bedding. Rats were housed individually, and the provided nesting material was assessed for complexity and structure. After an overnight period, photographs were taken to document nest construction, and the remaining amount of nesting material was measured. Animals received recombinant NRG1β1 (50 pg g⁻¹, i.p.) once daily for 9 consecutive days.

### Social transmission of food preference (STFP) test

The STFP test was conducted following procedures described in previous studies 31. The test was performed in both male and female rats following food habituation. Prior to STFP, all rats were food-deprived for 24 h. A demonstrator rat was separated and allowed to consume cued food (basil-flavored chow) for 1 h, while subject rats were provided standard chow for the same period. Before the STFP task, olfactory function was assessed using the buried cookie test. Each subject rat was placed in its home cage, and an Oreo cookie was buried 1 cm beneath the bedding. The location of the cookie was systematically varied across trials to avoid place preference. Rats were allowed 15 min to locate the cookie, and the latency to retrieval was recorded. For the STFP task, a demonstrator rat was introduced into the testing chamber and placed in the center zone. The subject rat was then allowed to freely interact with the demonstrator for 30 min, during which the interaction duration was recorded. To evaluate food preference, cued food was placed in the left zone and novel food (cinnamon-flavored chow) in the right zone. The subject rat was placed in the center zone and allowed to freely explore for 90 min. The time spent in each zone and the amount of food consumed were measured. Animals received recombinant NRG1β1 (50 pg g⁻¹, i.p.) once daily for 9 consecutive days**.** The apparatus was thoroughly cleaned with 70% ethanol between sessions.

### Prepulse inhibition (PPI) test

The PPI of the acoustic startle response was assessed using startle chambers. Rats were trained for 4-7 days by being placed in clear Plexiglas holding cylinders for 7 min, four times per day. A continuous 65 dB white noise was used throughout habituation and test sessions. During habituation, rats were presented with 29 startle trials (40 ms, 120 dB pulses) at 20-s intervals. During the test session, rats were presented with three trial types: (i) startle trials (40 ms, 120 dB pulses), (ii) prepulse + startle trials consisting of a 20 ms prepulse (68, 71, or 77 dB) followed 100 ms later by a 120 dB pulse, and (iii) no-stimulus trials. Each trial type was presented 10 times, with an average inter-trial interval of 15 s. Animals received recombinant NRG1β1 (50 pg g⁻¹, i.p.) once daily for 14 consecutive days. PPI was calculated as the percentage reduction of the startle response using the formula:

PPI (%) = 100 - [(startle amplitude after prepulse + pulse) / (startle amplitude after pulse only) × 100]

### Cross-assay behavioral integration and quadrant classification

To ensure reproducible and interpretable classification of behavioral phenotypes while avoiding arbitrary thresholds, we adopted a distribution-based approach. This approach is consistent with commonly used strategies in behavioral phenotyping that account for inter-individual variability 32,33, and was applied to cross-assay integration of impulsivity/disinhibition-related measures using bivariate plots combining EPM, CAT, and vCAT data.

For each behavioral parameter, the mean ± SD of the SAL group was used to define the normative range. Animals falling within this range were classified as normal, whereas those exceeding this range were classified as exhibiting impulsivity for the corresponding behavioral measure. Animals exceeding the defined range in both measures were categorized as impulsive in the integrated analysis. Importantly, this classification was based on distribution-based criteria derived from the SAL group rather than on arbitrary predefined cut-off values. In addition, the proportion of animals assigned to each category was calculated across the entire cohort, and these distributions were visualized using pie charts for each experimental group.

### Statistical analysis

Statistical analyses were performed using GraphPad Prism (version 10.6.0). Data are presented as mean ± SEM. Each experiment was conducted using independent biological replicates. Statistical significance between two groups was assessed using unpaired or paired Student's *t*-tests, as appropriate. For comparisons involving more than two groups, one-way ANOVA followed by Tukey's post hoc test was used. Normality of data distribution was evaluated using the D'Agostino-Pearson, Anderson-Darling, Shapiro-Wilk, and Kolmogorov-Smirnov tests.

For the PPI test, prepulse intensity was treated as a within-subject factor and analyzed using repeated-measures ANOVA to assess main and interaction effects. For the three-chamber test, within-group comparisons (e.g., empty vs. stranger or familiar vs. novel) were analyzed using paired *t*-tests, whereas between-group differences in interaction indices were assessed using one-way ANOVA followed by Tukey's multiple-comparisons test.

Statistical analyses were performed separately for male and female groups, and no pooled analysis across sexes was conducted. A *p*-value of < 0.05 was considered statistically significant.

## Results

### Cleaved NRG1 and its processing enzymes are reduced in the VPA rat model, with attenuated ErbB4 signaling and a transient early developmental delay

**Figure 1:**
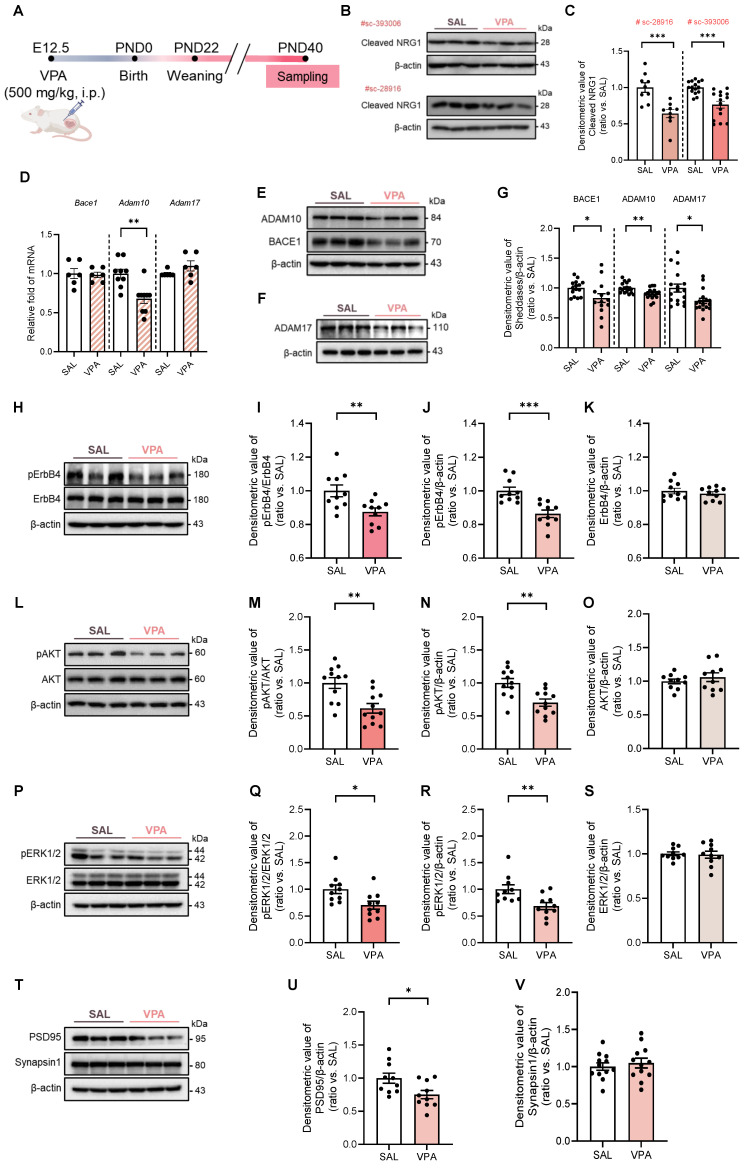
** Impaired NRG1 cleavage and diminished ErbB4 signaling with selective PSD95 reduction in the VPA rat model.** (**A**) Experiment schematic showing maternal VPA (500 mg kg⁻¹, i.p.) at E12.5. (**B**) Representative immunoblot of cleaved NRG1 detected with antibody sc-393006 and sc-28916. (**C**) Quantification shows reduced cleaved NRG1 with independent antibody in the VPA model. *n* = 9 per group in sc-28916; *n* = 15 per group in sc-393006. (**D**) Sheddases transcripts—* Bace1, Adam10, and Adam17*—mRNA levels. *Adam10* mRNA shows a modest reduction and *Bace1, Adam17* is unchanged. *n* = 6 per group in *Bace1; n* = 9 per group in *Adam10*; *n* = 6 per group in *Adam17*. (**E-F**) Representative immunoblot of BACE1, ADAM10 (**E**), and ADAM17 (**F**). (**G**) Quantification shows decreased BACE1, ADAM10, and ADAM17 protein. *n* = 15 per group in BACE1; *n* = 17 per group in ADAM10;* n* = 17 per group in ADAM17. (**H**) Representative immunoblots for pErbB4 and total ErbB4 with β-actin loading control. (**I**) Quantification of pErbB4/ErbB4 shows a decrease in the VPA model. (**J**) Quantification of pErbB4/β-actin shows a decrease. (**K**) ErbB4/β-actin is unchanged. *n* = 10 per group. (**L**) Representative immunoblots for pAKT and total AKT. (**M**) Quantification of pAKT/AKT shows a decrease. (**N**) Quantification of pAKT/β-actin shows a decrease. *n* = 11 per group. (**O**) AKT/β-actin is unchanged. *n* = 10 per group. (**P**) Representative immunoblots for pERK1/2 and total ERK1/2. (**Q**) Quantification of pERK1/2/ERK1/2 shows a decrease. (**R**) Quantification of pERK1/2/β-actin shows a decrease. (**S**) ERK1/2/β-actin is unchanged. *n* = 10 per group. (**T**) Representative immunoblots for PSD95 and Synapsin1. (**U**) Quantification shows PSD95 reduction. *n* = 10 per group. (**V**) Synapsin1 is preserved. *n* = 12 per group. Bars indicate mean ± SEM with individual animals shown; densitometry is normalized to β-actin and scaled to the SAL mean. Statistics: Two-sided Student's *t* test (**C-V**). Antibody catalog numbers are indicated above blots. *p < 0.05, **p < 0.01, and ***p < 0.001.

We first characterized early developmental trajectories in the VPA rat model of ASD, generated by a single maternal injection at E12.5 (500 mg kg⁻¹, i.p.) (Fig. 1A). Relative to controls, VPA-exposed offspring exhibited transient delays in physical maturation: body weight was reduced from PND14-35, righting reflex latencies were prolonged at PND3-4, and eye opening was delayed at PND12-13, converging with controls by PND15 (Supplementary Fig. S1A-G).

Biochemical profiling revealed a marked reduction in cleaved NRG1. This decrease was consistently observed using two independent antibodies (Fig. 1B, C), whereas total *Nrg1* mRNA levels and RNA-seq analysis remained unchanged (Supplementary Fig. S2A, B).

The transcripts were unchanged for *Adam17* and *Bace1* and only modestly reduced for *Adam10* (Fig. 1D), whereas principal sheddases—BACE1, ADAM10, and ADAM17—were also decreased at the protein level (Fig. 1E-G). These data indicate predominantly post-transcriptional or proteostatic impairment of NRG1 processing rather than broad transcriptional repression.

Downstream signaling mirrored these upstream alterations. pErbB4 was reduced while total ErbB4 remained (Fig. 1H-K). Similarly, pAKT/AKT and pERK1/2/ERK1/2 ratios were decreased without changes in total AKT (Fig. 1L-O) or ERK1/2 (Fig. 1P-S). In bulk hippocampal lysates, PSD95 protein was reduced whereas Synapsin1 remained unchanged (Fig. 1T-V), suggesting a preferential postsynaptic vulnerability at this stage. Collectively, these findings establish that the VPA model during adolescence exhibits impaired NRG1 processing, downregulation of ADAM10/17 and BACE1, attenuated ErbB4-AKT/ERK1/2 signaling, and early postsynaptic-predominant alterations.

### NRG1 mitigates repetitive and perseverative behaviors in the VPA rat model without altering locomotion

**Figure 2:**
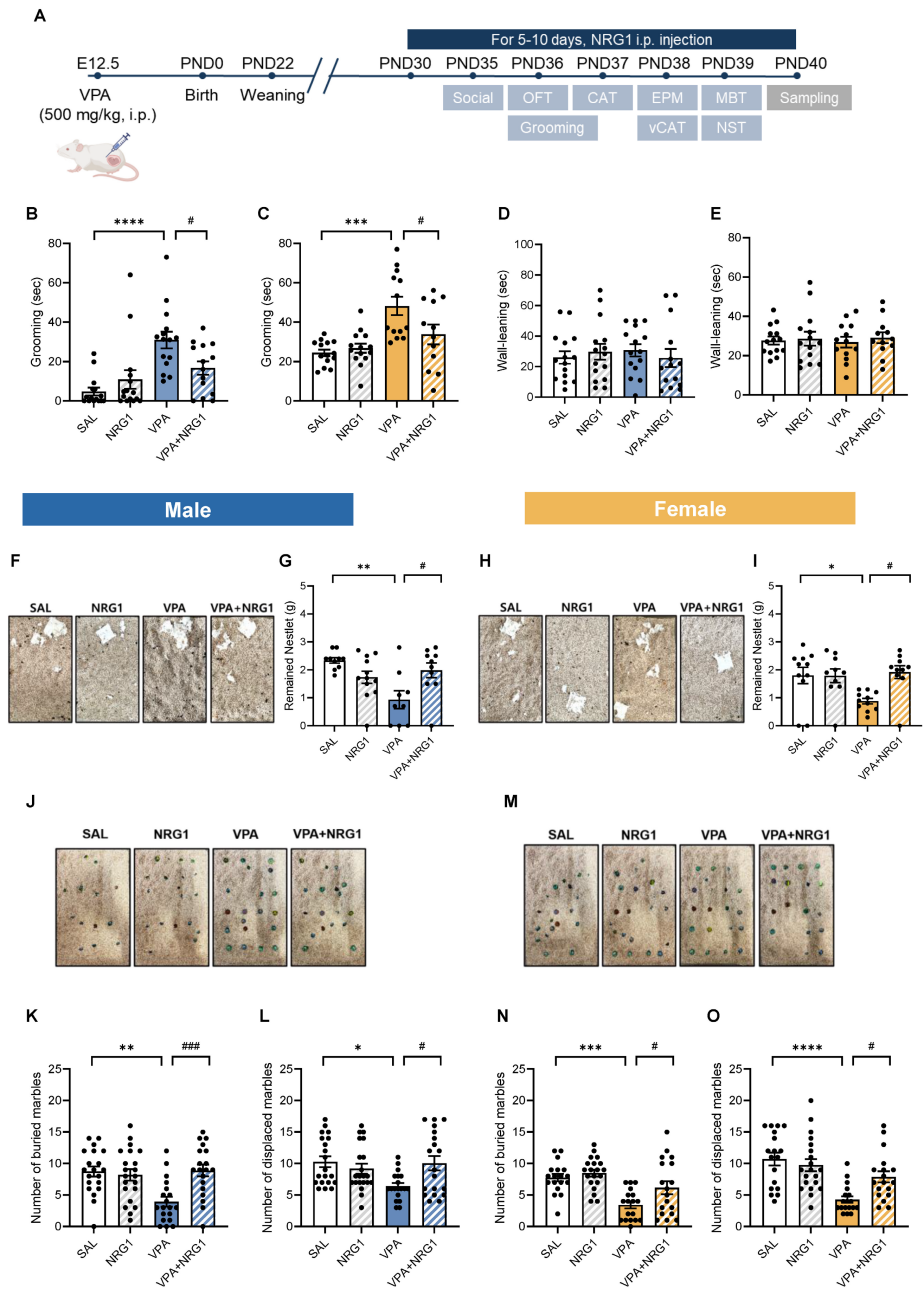
** NRG1 ameliorates repetitive/perseverative behaviors in the VPA rat model.** (**A**) Experimental timeline showing maternal VPA (500 mg kg⁻¹, i.p.) at E12.5. (**B**) Male grooming time is increased in the VPA model and reduced by NRG1. *n* = 15 rats in SAL;* n* = 15 rats in NRG1;* n* = 15 rats in VPA;* n* = 14 rats in VPA+NRG1. (**C**) Female grooming time shows the same pattern. (**D**) Male wall-leaning time shows no group difference.* n* = 14 rats in SAL;* n* = 14 rats in NRG1;* n* = 13 rats in VPA;* n* = 12 rats in VPA+NRG1. (**E**) Female wall-leaning time shows no group difference. (**F**) Representative nestlet images (male). (**G**) Male remaining nestlet mass is reduced in the VPA model and restored by NRG1.* n* = 10 rats in SAL;* n* = 12 rats in NRG1;* n* = 9 rats in VPA;* n* = 10 rats in VPA+NRG1. (**H**) Representative nestlet images (female). (**I**) Female remaining nestlet mass shows the same rescue by NRG1.* n* = 11 rats in SAL;* n* = 10 rats in NRG1;* n* = 11 rats in VPA;* n* = 10 rats in VPA+NRG1. (**J**) Representative marble burying images (male). (**K**) Male number of buried marbles is decreased in the VPA model and rescued by NRG1. (**L**) Male number of displaced marbles is decreased in the VPA model and rescued by NRG1. *n* = 20 rats in SAL;* n* = 21 rats in NRG1;* n* = 19 rats in VPA;* n* = 19 rats in VPA+NRG1. (**M**) Representative marble burying images (female). (**N**) Female number of buried marbles is decreased in the VPA model and rescued by NRG1. (**O**) Female number of displaced marbles is decreased in the VPA model and rescued by NRG1. *n* = 18 rats in SAL;* n* = 20 rats in NRG1;* n* = 18 rats in VPA;* n* = 18 rats in VPA+NRG1. Bars denote means ± SEM with individual animals shown. Statistics: One-way ANOVA followed by Tukey's multiple-comparisons test within sex (**B-E, G, I, K, L, N, O**). *p < 0.05, **p < 0.01, ***p < 0.001, and ****p < 0.0001.

Consistent with previous reports, prenatal VPA exposure recapitulated ASD-like behavioral phenotypes in our study 3,4, which were subsequently examined across multiple behavioral domains. Adolescent VPA-exposed offspring received daily i.p. injections of recombinant NRG1β1 for 5-10 consecutive days prior to behavioral testing (Fig. 2A). VPA animals displayed increased self-grooming, which was normalized by NRG1 treatment in both sexes (Fig. 2B, C). Wall-leaning time did not differ among groups (Fig. 2D, E).

In the nestlet shredding test, VPA rats shredded more material (resulting in reduced remaining nestlet mass), whereas NRG1 restored this measure toward control levels in both males and females (Fig. 2F-I). In the marble burying and displacement tests, VPA animals buried and displaced fewer marbles than controls, and these measures were restored by NRG1 treatment (Fig. 2J-O). Open-field parameters—including zone occupancy, total distance, velocity, and transitions—were unchanged across groups (Supplementary Fig. S4A-O), indicating that these behavioral effects are not attributable to alterations in general locomotor activity.

### NRG1 alleviates disinhibition- and impulsivity-like behaviors in the VPA rat model, as shown by convergent results from CAT and vCAT

**Figure 3:**
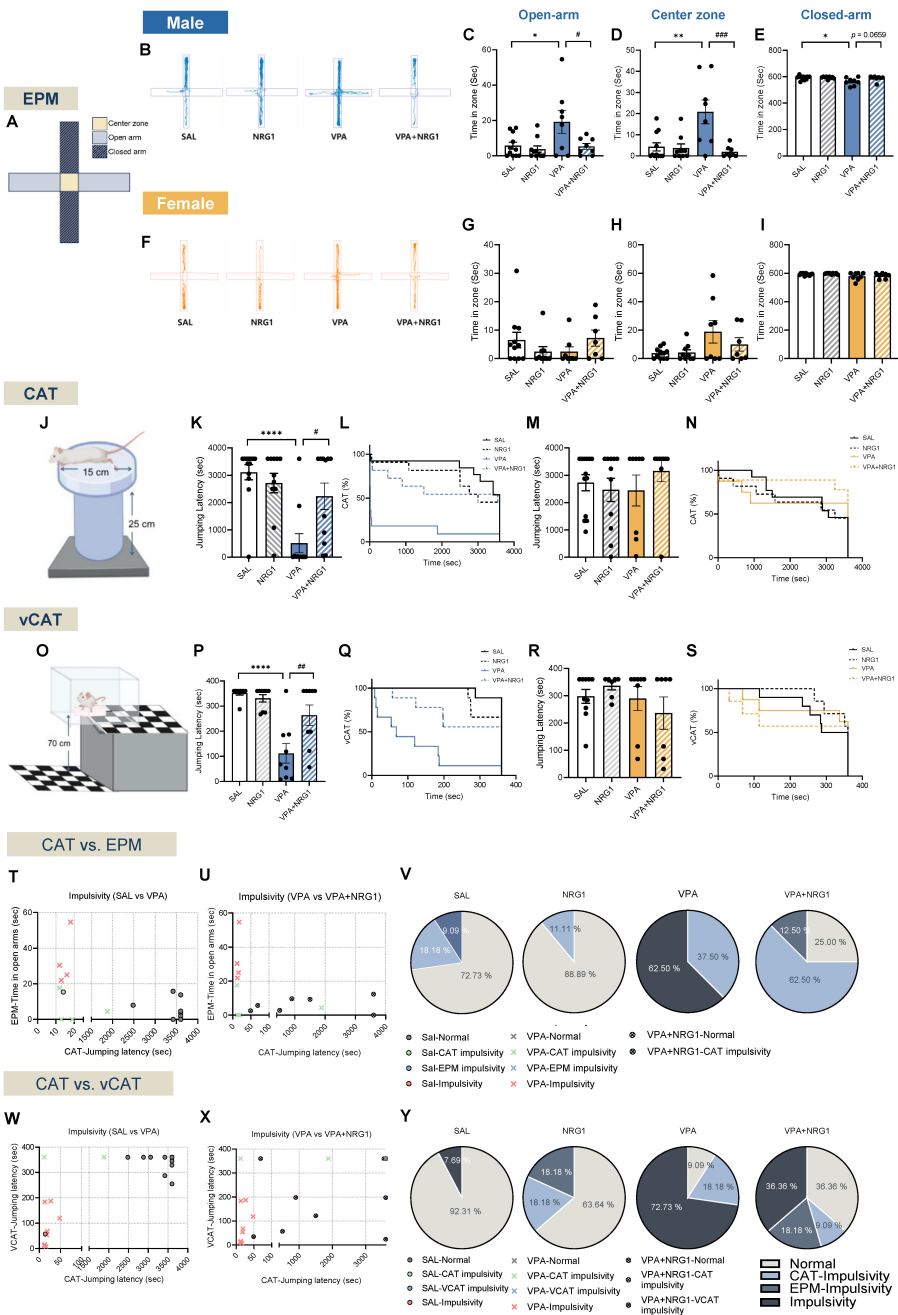
** NRG1 normalizes disinhibition-/impulsivity-related measures across EPM, CAT, and vCAT and decreases the proportion of impulsive animals in VPA rat model.** (**A**) EPM schematic with center zone, open and closed-arms. (**B**) Representative EPM tracks for males across groups. (**C**) Male open-arm time is increased in the VPA model and reduced by NRG1. (**D**) Male center zone time is increased in the VPA model and reduced by NRG1. (**E**) Male closed-arm time is reduced in the VPA model with a trend toward normalization by NRG1. *n* = 11 rats in SAL;* n* = 9 rats in NRG1;* n* = 8 rats in VPA;* n* = 8 rats in VPA+NRG1. (**F**) Representative EPM tracks for females across groups. (**G**) Female open-arm time shows no group difference. (**H**) Female center zone time shows no group difference. (**I**) Female closed-arm time shows no group difference. *n* = 11 rats in SAL;* n* = 9 rats in NRG1;* n* = 8 rats in VPA;* n* = 7 rats in VPA+NRG1. (**J**) CAT schematic. (**K**) Male CAT jumping latency is shortened in the VPA model and prolonged by NRG1. (**L**) Male CAT Kaplan-Meier curves show earlier jumping in VPA and a right-shift with NRG1. *n* = 13 rats in SAL;* n* = 11 rats in NRG1;* n* = 11 rats in VPA;* n* = 11 rats in VPA+NRG1. (**M**) Female CAT jumping latency shows no group difference. (**N**) Female CAT Kaplan-Meier curves show minimal separation. (**O**) vCAT schematic. (**P**) Male vCAT jumping latency is shortened in the VPA model and prolonged by NRG1. (**Q**) Male vCAT Kaplan-Meier curves show earlier jumping in VPA and a right-shift with NRG1.* n* = 9 per group. (**R**) Female vCAT jumping latency shows no group difference. (**S**) Female vCAT Kaplan-Meier curves show minimal separation. *n* = 10 rats in SAL;* n* = 7 rats in NRG1;* n* = 8 rats in VPA;* n* = 7 rats in VPA+NRG1. (**T**) Male scatter of EPM open-arm time versus CAT jumping latency for SAL versus VPA. (**U**) Male scatter of EPM open-arm time versus CAT jumping latency for VPA versus VPA+NRG1. (**V**) Male pie charts showing proportions classified as Normal, CAT-impulsivity, EPM-impulsivity or Dual-impulsivity across groups. (**W**) Male scatter of vCAT jumping latency versus CAT jumping latency for SAL versus VPA. (**X**) Male scatter of vCAT jumping latency versus CAT jumping latency for VPA versus VPA+NRG1. (**Y**) Male pie charts showing category proportions for the CAT-vCAT pairing. Bars denote means ± SEM with individual animals shown; each symbol represents one animal. Classification rules and thresholds for categories were predefined in the Methods section. Statistics: Fisher's exact test with Holm-Bonferroni correction for differences in category proportions; One-way ANOVA followed by Tukey's multiple-comparisons test within sex (**C-I, K, M, P, R**); log-rank (Mantel-Cox) tests for Kaplan-Meier curves (**L, N, Q, S**). *p < 0.05, **p < 0.01, ***p < 0.001, and ****p < 0.0001.

In the EPM (Fig. 3A), male VPA rats spent more time in the open-arms and center zone, with reduced closed-arm time compared to controls. NRG1 treatment shifted these metrics toward control levels reducing open/center occupancy and a trend towards greater closed-arm time (Fig. 3B-E). Female groups showed minimal differences (Fig. 3F-I). Because increased open-arm exploration can reflect either reduced anxiety or behavioral disinhibition, the male EPM pattern was interpreted alongside impulsivity-specific paradigms.

In the CAT (Fig. 3J) and its visual variant (vCAT; Fig. 3O), male VPA animals exhibited shorter latencies to jump, reflecting impulsive escape tendencies. NRG1 prolonged these latencies across both bar-plot summaries and Kaplan-Meier survival curves (Fig. 3K, L; Supplementary Video 1 for CAT; Fig. 3P, Q for vCAT). Females again showed little differences (Fig. 3M, N for CAT; Fig. 3R, S for vCAT). Because open-field metrics were unchanged, these effects reflect domain-specific modulation of impulsivity rather than motoric confounds.

Bivariate plots combining CAT-EPM and CAT-vCAT readouts placed male VPA rats within predefined “impulsivity/disinhibited” quadrants, whereas NRG1 shifted the distribution toward the normative range (Fig. 3T-V for CAT-EPM, 3W-Y for CAT-vCAT in males). Corresponding pie charts confirmed that NRG1 reduced the impulsive fraction and increased the normal fraction in males, with minimal effects in females (Supplementary Fig. S5A-C for CAT and S5D-F for vCAT in females). Together, these findings demonstrate that the male-biased pattern of elevated open-arm exploration and cliff jumping in the VPA model reflects impulsivity/disinhibition rather than reduced anxiety, and NRG1 normalizes these behaviors across assays.

### NRG1 rescues male-specific deficits in sociability and social novelty in the VPA rat model

**Figure 4:**
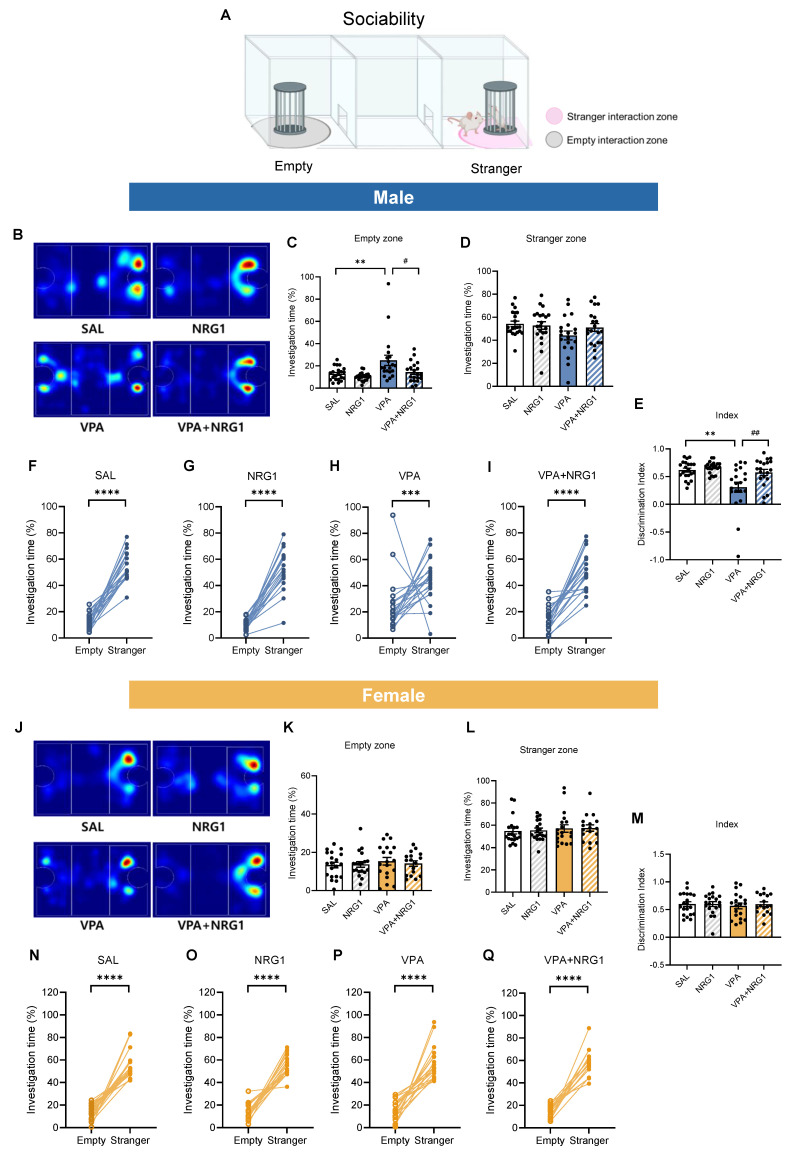
** NRG1 restores male sociability in the VPA rat model.** (**A**) Sociability schematics with empty and stranger interaction zones. (**B**) Representative heat maps of exploration (male). (**C**) Male empty-zone time is increased in VPA and reduced by NRG1. (**D**) Male stranger-zone time shows limited decrease in VPA and improvement with NRG1. (**E**) Male discrimination index (stranger vs empty) is reduced in VPA and improved by NRG1. (**F-I**) Within-group paired comparisons show stranger preference in SAL, NRG1, and VPA+NRG1, with a blunted effect in VPA (male). *n* = 21 rats in SAL;* n* = 21 rats in NRG1;* n* = 20 rats in VPA;* n* = 20 rats in VPA+NRG1. (**J**) Representative heat maps of exploration (female). (**K-M**) Female empty-/stranger-zone times and discrimination index show minimal group differences; stranger preference is preserved (**N-Q**). *n* = 20 rats in SAL;* n* = 19 rats in NRG1;* n* = 19 rats in VPA;* n* = 17 rats in VPA+NRG1. Bars denote means ± SEM with individual animals shown; each line in paired plots represents one animal. Statistics: One-way ANOVA followed by Tukey's multiple-comparisons test for between-group bars (**C-E, K-M**); paired two-sided t tests within group for empty vs stranger (F-G, I, O-Q); Mann-Whitney test (two-tailed) (**H, N**). *p < 0.05, **p < 0.01, and ****p < 0.0001.

In the three-chamber sociability test (Fig. 4A), male VPA rats spent more time in the empty chamber and less time with the social target, yielding a reduced discrimination index. NRG1 treatment shifted these metrics toward control values (Fig. 4B-E). Within-group analyses confirmed robust stranger preference in control, NRG1, and VPA+NRG1 groups, but a blunted effect in VPA (Fig. 4F-I). Females exhibited minimal between-group differences (Fig. 4J-M), and within-group paired analyses verified intact social preference across all female conditions (Fig. 4N-Q).

**Figure 5:**
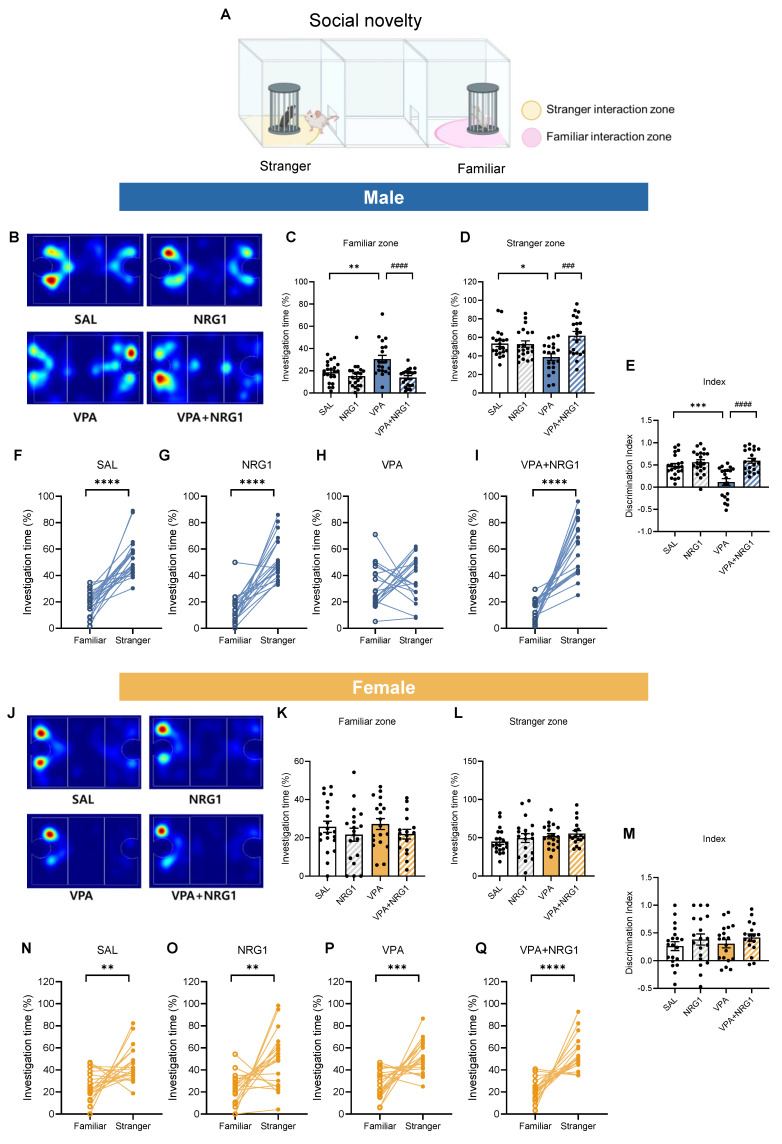
** NRG1 restores male social-novelty recognition in the VPA rat model.** (**A**) Social-novelty schematic with familiar and novel-stranger interaction zones. (**B**) Representative heat maps of exploration (male). (**C**) Male familiar-zone time is increased in VPA and reduced by NRG1. (**D**) Male novel-stranger zone time is decreased in VPA and increased by NRG1. (**E**) Male discrimination index (novel vs familiar) is reduced in VPA and restored by NRG1. (**F-I**) Within-group paired comparisons show intact novelty preference in SAL and NRG1, loss of preference in VPA, and restoration in VPA+NRG1 (male). *n* = 21 rats in SAL;* n* = 21 rats in NRG1;* n* = 20 rats in VPA;* n* = 20 rats in VPA+NRG1. (**J**) Representative heat maps of exploration (female). (**K-M**) Female familiar-/novel-stranger times and discrimination index show minimal group differences; novelty preference is preserved (**N-Q**). *n* = 20 rats in SAL;* n* = 19 rats in NRG1;* n* = 19 rats in VPA;* n* = 17 rats in VPA+NRG1. Bars denote means ± SEM with individual animals shown; each line in paired plots represents one animal. Statistics: One-way ANOVA followed by Tukey's multiple-comparisons test for between-group bars (**C-E, K-M**); paired two-sided t tests within group for familiar vs novel stranger (**F, H-I, N-Q**); Mann-Whitney test (two-tailed) (G). *p < 0.05, **p < 0.01, ***p < 0.001, and ****p < 0.0001.

In the social novelty phase (Fig. 5A), male VPA rats failed to prefer the novel conspecific, spending more time in the familiar zone and showing a lower discrimination index. NRG1 restored novelty preference (Fig. 5B-E), with within-group analyses confirming no preference in VPA and robust novelty preference after NRG1 (Fig. 5F-I). Females again showed preserved novelty preference (Fig. 5J-Q). Together, these data indicate that male-biased social deficits in the VPA model are rescued by NRG1 without locomotor confounds (Supplementary Fig. S4).

### NRG1 restores social-olfactory learning and sensorimotor gating in male, but not female VPA rats

**Figure 6:**
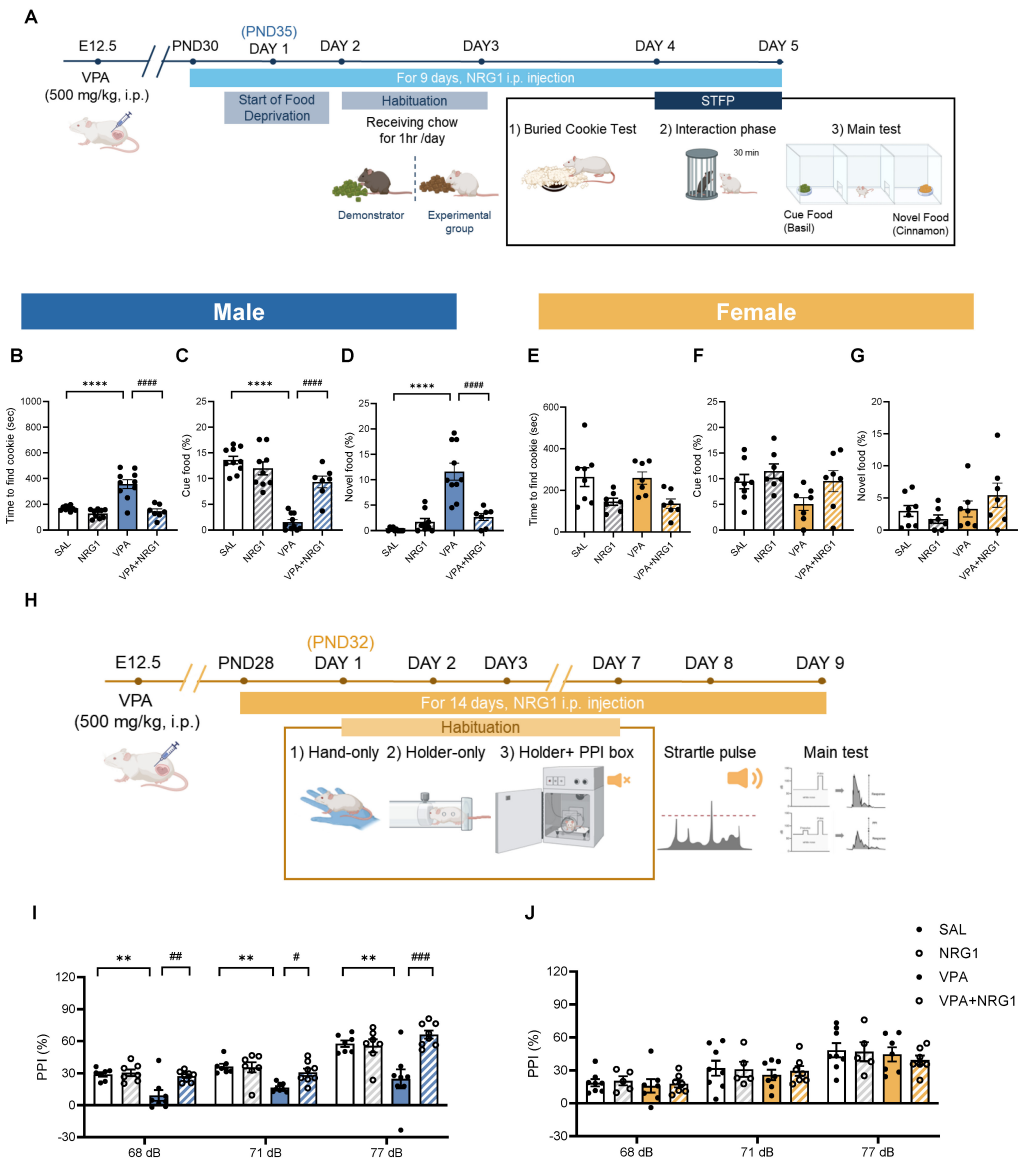
** NRG1 restores social-olfactory preference and rescues sensorimotor gating in male VPA rats.** (**A**) STFP timeline showing food deprivation/habituation, daily NRG1 administration, buried-cookie test, demonstrator interaction, and main test comparing cue and novel food. (**B**) Male buried-cookie latency is increased in VPA and reduced by NRG1. (**C**) Male cue-food consumption is decreased in VPA and increased by NRG1. (**D**) Male novel-food consumption is increased in VPA and reduced by NRG1. *n* = 10 rats in SAL;* n* = 9 rats in NRG1;* n* = 10 rats in VPA;* n* = 7 rats in VPA+NRG1. (**E**) Female buried-cookie latency shows little or no group difference. (**F**) Female cue-food consumption shows little or no group difference. (**G**) Female novel-food consumption shows little or no group difference. *n* = 8 rats in SAL;* n* = 7 rats in NRG1;* n* = 7 rats in VPA;* n* = 7 rats in VPA+NRG1. (**H**) PPI timeline and procedure (habituation; startle and prepulse presentations). (**I**) Male PPI is reduced in VPA at multiple prepulse intensities and rescued by NRG1. *n* = 7 rats in SAL;* n* = 7 rats in NRG1;* n* = 7 rats in VPA;* n* = 8 rats in VPA+NRG1. (**J**) Female PPI shows no group difference across prepulse intensities. *n* = 8 rats in SAL;* n* = 5 rats in NRG1;* n* = 7 rats in VPA;* n* = 7 rats in VPA+NRG1. Bars denote mean ± SEM with individual animals shown. Statistics: One-way ANOVA followed by Tukey's multiple-comparisons test within sex for STFP (**B-G**) and PPI (**I-J**); PPI (%) was computed as *100 × [(startle-alone - startle-prepulse)/startle-alone]*. *p < 0.05, **p < 0.01, ***p < 0.001, and ****p < 0.0001.

To further examine social-cognitive domains, we assessed social-olfactory learning and sensorimotor gating. In the STFP test (Fig. 6A-G), male VPA rats exhibited longer latencies to locate the buried cookie (Fig. 6B) and reduced preference for the demonstrator-cued food, coupled with greater consumption of novel food (Fig. 6C, D). NRG1 treatment restored performance across all measures. Female groups showed minimal or no changes (Fig. 6E-G).

In the PPI test, which measures sensorimotor gating (Fig. 6H-J), male VPA animals showed reduced PPI across prepulse intensities, whereas NRG1 normalized these responses (Fig. 6I). Females again displayed little differences (Fig. 6J). Together, these results indicate that prenatal VPA exposure impairs both higher-order social learning and lower-level sensorimotor filtering processes, and that NRG1 treatment restores behavioral function across these levels.

Collectively, these results indicate that NRG1 corrects male-biased impairments in social-olfactory learning and sensorimotor gating in the VPA model.

### NRG1 restores hippocampal ErbB4-AKT/ERK1/2 signaling without altering sheddase transcripts

**Figure 7:**
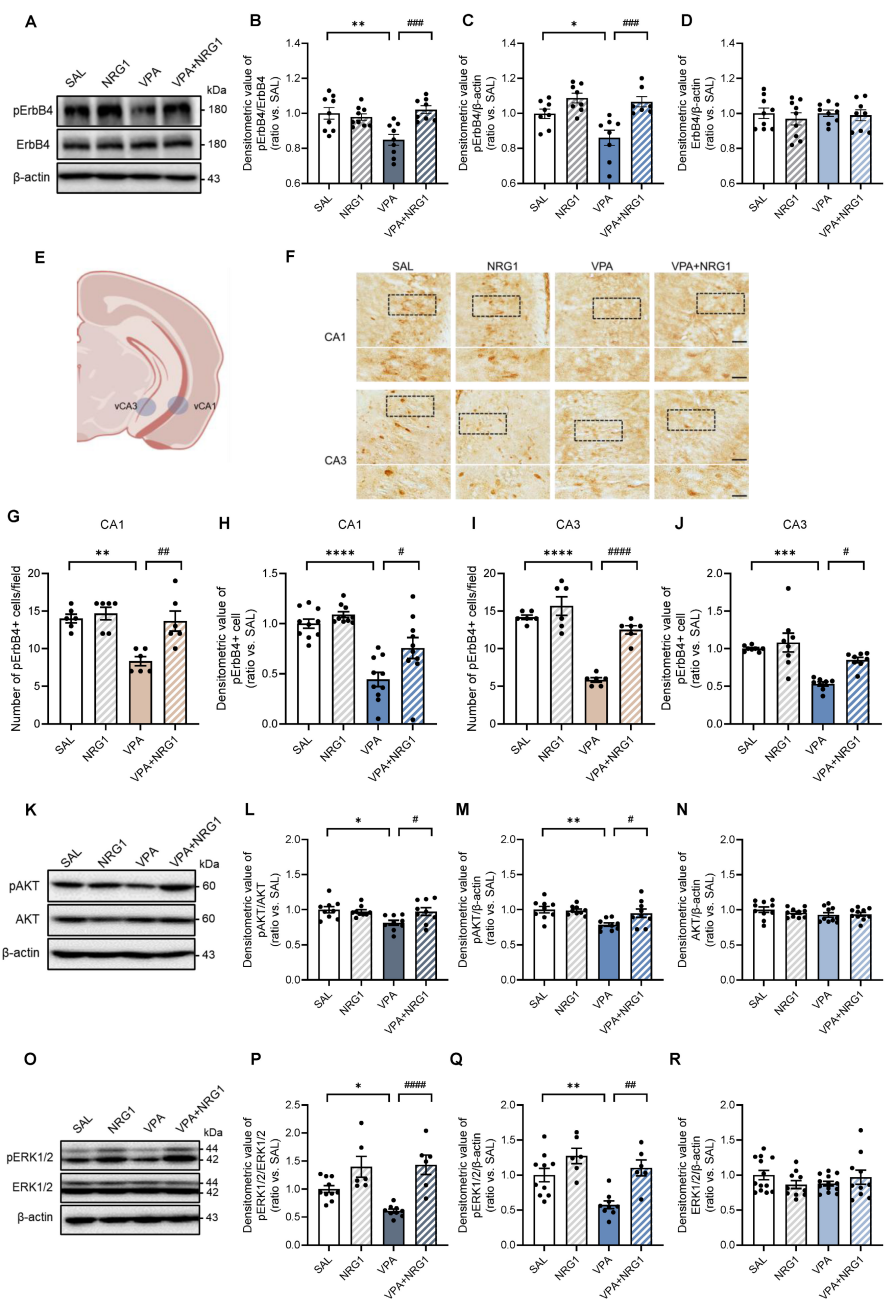
** NRG1 restores hippocampal ErbB4-AKT/ERK1/2 signaling in male VPA rats.** (**A**) Representative immunoblots for pErbB4 and ErbB4 in male hippocampus with β-actin loading control. (**B**) pErbB4/ErbB4 is reduced in VPA and restored by NRG1. (**C**) pErbB4/β-actin is decreased in VPA and restored by NRG1. (**D**) ErbB4/β-actin is unchanged. *n* = 9 rats in SAL;* n* = 9 rats in NRG1;* n* = 9 rats in VPA;* n* = 8 rats in VPA+NRG1. (**E**) Representative brain region scheme with vCA1 and vCA3. (**F**) pErbB4 immunohistochemistry in male hippocampal vCA1 and vCA3 (boxed regions). Scale bar, 50 μm; enlargement 25 μm. (**G**) vCA1 quantification shows a reduced number of pErbB4^+^ cells in VPA, restored by NRG1. *n* = 6 per group. (**H**) vCA1 quantification shows a reduced signal intensity in VPA, restored by NRG1. *n* = 10 per group. (**I**) vCA3 quantification shows a reduced number of pErbB4^+^ cells in VPA and rescued by NRG1. *n* = 6 per group. (**J**) vCA3 quantification shows a reduced signal intensity in VPA and rescued by NRG1. *n* = 8 per group. (**K**) Representative immunoblots for pAKT and AKT in male hippocampus. (**L**) pAKT/AKT is decreased in VPA and normalized by NRG1. (**M**) pAKT/β-actin is decreased in VPA and normalized by NRG1.* n* = 9 per group. (**N**) AKT/β-actin is unchanged. *n* = 10 per group. (**O**) Representative immunoblots for pERK1/2 and ERK1/2 in male hippocampus. (**P**) pERK1/2/ERK1/2 is decreased in VPA and restored by NRG1. (**Q**) pERK1/2/β-actin is decreased in VPA and restored by NRG1.* n* = 10 rats in SAL;* n* = 6 rats in NRG1;* n* = 9 rats in VPA;* n* = 6 rats in VPA+NRG1. (**R**) ERK1/2/β-actin is unchanged. *n* = 10 rats in SAL;* n* = 7 rats in NRG1;* n* = 10 rats in VPA;* n* = 7 rats in VPA+NRG1. Bars denote means ± SEM with individual animals shown; densitometry is normalized to β-actin and scaled to the SAL mean. Statistics: One-way ANOVA followed by Tukey's multiple-comparisons test (**B-D, G-J, L-N, P-R**). *p < 0.05, **p < 0.01, ***p < 0.001, and ****p < 0.0001.

Because behavioral abnormalities and NRG1 responsiveness were male-biased, biochemical analyses were confined to males. In hippocampal lysates, pErbB4 was reduced in VPA and restored by NRG1, while total ErbB4 was unchanged (Fig. 7A-D). Immunohistochemistry revealed fewer and less intensely stained interneuron-like pErbB4^+^ cells in VPA animals, both of which were rescued by NRG1 (Fig. 7F-J). Downstream, pAKT/AKT and pERK1/2/ERK1/2 ratios were lowered by VPA and normalized by NRG1, whereas total AKT (Fig. 7K-N) and ERK1/2 (Fig. 7O-R) remained stable. Consistent with a post-transcriptional mechanism, qPCR showed no changes in* Adam10*, *Adam17*, or *Bace1* transcripts between VPA and VPA+NRG1 groups (Supplementary Fig. S6A-C). Together, these data indicate that NRG1 reactivates the hippocampal ErbB4 signaling cascade in male VPA rats without altering sheddase gene expression.

### NRG1 restores hippocampal synaptic scaffolding and spine maturity in male VPA rats

**Figure 8:**
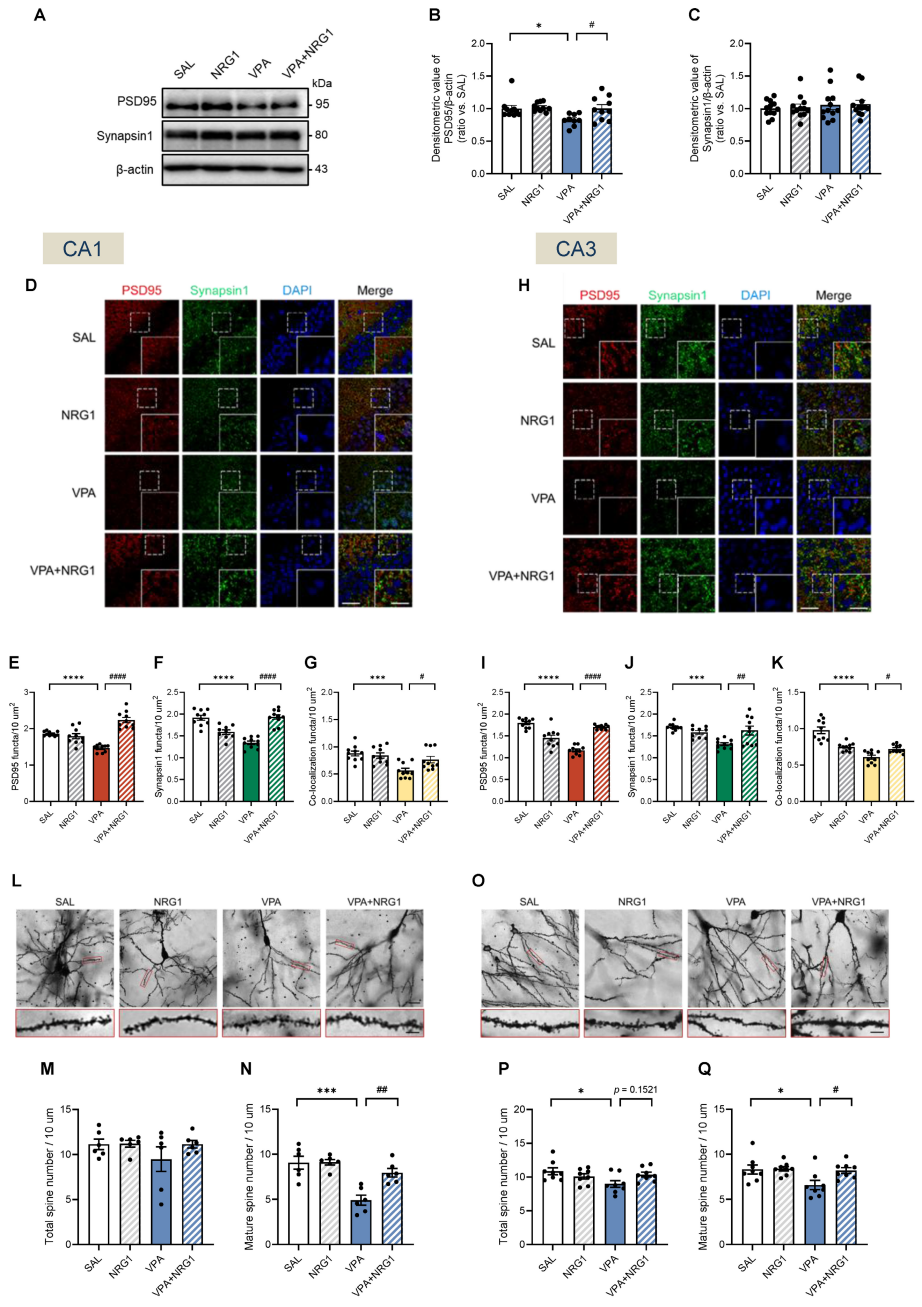
** NRG1 normalizes postsynaptic scaffolding and spine maturity in the male VPA hippocampus.** (**A**) Representative immunoblots for PSD95 and Synapsin1 with β-actin loading control. (**B**) PSD95/β-actin is decreased in VPA and restored by NRG1. *n* = 10 rats in SAL;* n* = 10 rats in NRG1;* n* = 9 rats in VPA;* n* = 10 rats in VPA+NRG1. (**C**) Synapsin1/β-actin is unchanged across groups. *n* = 12 per group. (**D-G**) vCA1 confocal images for PSD95 (red), Synapsin1 (green), DAPI (blue) and merge (**D**) with quantification of PSD95 puncta number (**E**), Synapsin1 puncta number (**F**) and PSD95-Synapsin1 co-localization (**G**); values are reduced in VPA and restored by NRG1. Scale bar, 50 μm; enlargement, 25 μm. (**H-K**) vCA3 confocal images (**H**) with quantification for PSD95 puncta number; *n* = 10 per group (**I**), Synapsin1 puncta number; *n* = 10 rats in SAL,* n* = 9 rats in NRG1,* n* = 10 rats in VPA,* n* = 10 rats in VPA+NRG1 (**J**) and co-localization; n = 10 per group. (**K**); VPA is reduced and NRG1 rescues. (**L**) Representative Golgi images and dendritic segments (vCA1). (**M**) vCA1 total spine density shows no group difference. (**N**) vCA1 mature spine density is reduced in VPA and increased by NRG1. *n* = 6 per group. (**O**) Representative Golgi images and dendritic segments (vCA3). (**P**) vCA3 total spine density shows a small reduction in VPA with partial normalization by NRG1. (**Q**) vCA3 mature spine density is reduced in VPA and increased by NRG1. *n* = 8 per group. Bars denote means ± SEM with individual animals shown; densitometry is normalized to β-actin and scaled to the SAL mean. Puncta densities and co-localization are expressed per µm²; spine classes follow a priori morphological criteria (Methods). The same β-actin blot is shown in Fig. 7A and Fig. 8A because the same quantified lysate samples were loaded in parallel gels, and the β-actin signal obtained from the 15 μg protein loading, which was within the appropriate exposure range, was used for normalization. Statistics: One-way ANOVA followed by Tukey's multiple-comparisons test (**B-C, E-G, I-K, M-N, P-Q**). *p < 0.05, **p < 0.01, ***p < 0.001, and ****p < 0.0001.

Given the male-selective behavioral phenotype and signaling rescue, synaptic assessments were performed in males. In whole hippocampal lysates, PSD95 protein was reduced in VPA and restored by NRG1, while Synapsin1 levels remained largely stable at this bulk level (Fig. 8A-C). Confocal immunostaining revealed reduced PSD95 and Synapsin1 puncta density, as well as diminished co-localization, both of which were normalized by NRG1 in ventral CA1 (vCA1) and CA3 (Fig. 8D-K). Golgi analysis further showed that total spine density was largely preserved in vCA1 and mildly reduced in vCA3, whereas mature spines were selectively reduced in VPA and restored by NRG1 across both regions (Fig. 8L-Q). Together with the signaling results (Fig. 7), these data indicate that NRG1 restores both pre- and postsynaptic scaffolding and dendritic spine maturation in the male hippocampus, consistent with engagement of ErbB4-AKT/ERK1/2 signaling.

## Discussion

NRG1-ErbB signaling has long been placed at the intersection of synapse biology and neuropsychiatric risk, yet its mechanistic status in ASD-relevant conditions remains unresolved. Here, we show that impaired NRG1 processing—together with reduced ADAM10/17 and BACE1 protein abundance—is associated with molecular and behavioral alterations in the prenatal VPA rat model. This deficit is accompanied by attenuated hippocampal ErbB4-AKT/ERK1/2 signaling and coordinated pre- and postsynaptic abnormalities, all of which are restored by NRG1 administered during adolescence. Importantly, these findings support an association between altered NRG1 processing and ASD-related phenotypes, rather than establishing a direct causal mechanism.

Although VPA-exposed animals showed reduced body weight during development, this phenotype is a well-established feature of the model and is not considered a nonspecific health impairment 3,4. Because key developmental milestones recovered over time and behavioral testing was conducted after this recovery, the observed behavioral phenotypes are unlikely to reflect global developmental delay.

Epidemiological cohorts consistently associate *in utero* VPA exposure with increased ASD risk 34,35, providing clinical grounding for this model. Our dataset captures core ASD-relevant domains and demonstrates that adolescent NRG1 normalizes multiple behavioral abnormalities in the VPA model. Notably, repetitive/perseverative phenotypes were improved in both sexes, whereas rescue effects in social, social-cognitive, and sensorimotor-gating domains were more evident in males. Interpretation of the EPM and cliff-avoidance tasks benefits from a multidimensional framework. Although increased open-arm exploration is often interpreted as reduced anxiety 22,36, it can also reflect disinhibition depending on circuit context 28,32,37. In our model, the combination of increased open-arm exploration and reduced jumping latency is inconsistent with anxiolysis and instead reflects impaired inhibitory control.

Given that NRG1-ErbB4 signaling regulates PV interneuron-mediated inhibitory control, disruption of this pathway may underlie the observed impulsivity and disinhibition phenotypes 13,21. This interpretation is further supported by cross-assay convergence, in which NRG1 treatment consistently shifted animals from an impulsive/disinhibited behavioral state toward the normative range. These findings suggest restoration of executive inhibitory gating rather than nonspecific suppression of activity.

In the repetitive/perseverative domain, prenatal VPA exposure produced a qualitative imbalance characterized by increased self-directed stereotypy (grooming, nestlet shredding) and reduced goal-directed digging behavior. Given the multidimensional nature of marble burying behavior, this reduction is unlikely to reflect a simple decrease in repetitive behavior but rather altered exploratory or goal-directed processes 38,39. NRG1 normalized both components without affecting locomotor activity. The absence of open-field differences further supports that these behavioral changes are not driven by motor deficits, but rather reflect altered behavioral organization and flexibility.

In the social domain, VPA-exposed males exhibited deficits in sociability and social novelty that were fully restored by NRG1. These paradigms are well-established measures of social behavior 40,41, and similar impairments have been reported in both VPA and NRG1 genetic-related models 4,42-46. The male-specific deficit pattern, in the absence of locomotor differences, suggests impaired social information processing rather than reduced motivation.

To further dissect social cognition, we integrated STFP and PPI paradigms. In males, VPA disrupted both social-olfactory learning and sensorimotor gating, both of which were restored by NRG1. The parallel impairment and rescue of STFP and PPI suggest that NRG1-ErbB4 signaling operates across hierarchical levels of neural processing, from early sensory filtering to higher-order social cognition. These findings support a model in which NRG1 modulates distributed circuit functions rather than isolated behavioral domains.

Although we did not directly assess brain penetration of systemically administered NRG1β1 in the present study, previous studies have demonstrated that peripheral NRG1β1 can cross the blood-brain barrier (BBB) and activate central signaling pathways 20-22. However, the present study cannot distinguish whether the observed effects arise from direct central nervous system (CNS) actions or indirect peripheral-to-central mechanisms. NRG1 has been implicated in immune regulation and neuroinflammatory signaling, including modulation of microglial activation and NF-κB-dependent pathways, and partial Nrg1 deletion has been reported to alter stress-related HPA-axis function and sensorimotor-gating phenotypes 47-49. Therefore, neuroimmune or endocrine pathways may have contributed, at least in part, to the observed effects, representing an important limitation of the present study.

At the molecular level, most NRG1 isoforms are synthesized as membrane precursors that require ectodomain shedding by ADAM10/17 and BACE1 to release the active ligand 50,51. In our model, reduced sheddase abundance and decreased cleaved NRG1, despite largely unchanged *Nrg1* transcripts, indicate predominantly post-transcriptional mechanisms affecting NRG1 processing that limits ligand availability. Consequently, ErbB4-AKT/ERK1/2 signaling is attenuated in the hippocampus, producing coordinated synaptic alterations. Exogenous NRG1 supplementation during adolescence restored ErbB4 phosphorylation, downstream signaling, and multidomain behavioral abnormalities, supporting a functional link between ligand availability and circuit regulation.

Although reduced pErbB4 immunoreactivity was observed in interneuron-like cells, these findings should be interpreted cautiously given the lack of cell-type-specific resolution. Rather than demonstrating definitive interneuron-specific effects, these data are more appropriately interpreted as reflecting circuit-level modulation of inhibitory signaling.

The coordinated recovery of PSD95, Synapsin1, and dendritic spine structure suggests restoration of synaptic organization. These findings are consistent with previous reports that NRG1-ErbB4 signaling regulates synaptic development through trans-synaptic mechanisms 15,19. However, we refrain from concluding direct restoration of E/I balance in the absence of electrophysiological evidence.

Circuit-level organization provides a mechanistic bridge linking molecular changes to behavior. NRG1 is primarily released from excitatory neurons, whereas ErbB4 is enriched in interneurons, allowing bidirectional regulation of synaptic input and inhibitory output 52-54. Our findings are consistent with an ErbB4-dependent circuit mechanism underlying behavioral normalization.

While ASD involves multiple brain regions, this study focused on the hippocampus due to its role in social memory and relevance to the behavioral paradigms employed. This region-specific focus represents a limitation, and future studies should examine other ASD-relevant circuits.

Adolescence represents a critical developmental window characterized by ongoing synaptic and inhibitory circuit maturation. This period provides a biologically relevant window for intervention and behavioral assessment 13,19,55. Notably, in the VPA model, this stage coincides with the emergence of ASD-relevant behavioral and circuit abnormalities 35, including E/I imbalance and PV interneuron dysfunction, which are key contributors to ASD-related phenotypes 56.

The observed male-biased effects should be interpreted cautiously. Rather than indicating baseline sex differences, these findings more likely reflect sex-dependent responsiveness to NRG1 treatment. This interpretation aligns with known sex differences in ASD prevalence and neurodevelopment 15,57,58.

In summary, our findings delineate a molecular-to-behavioral cascade in which impaired NRG1 processing is associated with reduced ErbB4 signaling and altered circuit function. NRG1 supplementation restores synaptic organization and behavioral phenotypes, supporting the NRG1-ErbB4 pathway as a functionally relevant mechanism in ASD-related neurodevelopmental dysfunction.

## Supplementary Material

Supplementary figures and video legend.

Supplementary video.

## Data Availability

All data supporting the findings of this study are available within the article and its Supplementary Information files. The RNA-seq dataset generated in this study is currently undergoing additional analyses and will be deposited in a public repository upon completion of these analyses. In the meantime, the data are available from the corresponding author upon reasonable request. Further information and requests for resources and reagents should be directed to and will be fulfilled by the Lead Contact (rswoo@eulji.ac.kr).

## Abbreviations

ASD: Autism spectrum disorder
BBB: Blood-brain barrier
BSA: Bovine serum albumin
CAT: Cliff avoidance test
CNS: Central nervous system
EPM: Elevated plus maze
E/I balance: Excitation-inhibition balance
i.p. injection: Intraperitoneal injection
MBT: Marble burying test
NRG1: Neuregulin 1
NST: Nest shredded test
OFT: Open field test
PBS: Phosphate-buffered saline
pErbB4: phosphorylated ErbB4
PFA: Paraformaldehyde
PND: Postnatal day
PPI: Prepulse inhibition
PSD95: Postsynaptic density protein 95
PV: Parvalbumin
qPCR: Quantitative real-time PCR
ROI: Regions of interest
RT: Room temperature
SD: Sprague-Dawley
STFP: Social transmission of food preference
TBS: Tris-buffered saline
VPA: Valproic acid
vCA: Ventral cornu ammonis
vCAT: Visual cliff avoidance test
