# Supplementary material for: Impaired Neuregulin 1 Processing is Associated with Synaptic and Behavioral Abnormalities in a Prenatal Valproic Acid Model of Autism
Source: Int J Biol Sci. 2026 May 18;22(10):5595–614. doi: 10.7150/ijbs.133137 (PMC13215460; doi:10.7150/ijbs.133137)
Supplement: Supplementary file 1 — Supplementary figures and video legend. [file ijbsv22p5595s1.pdf]

Impaired Neuregulin 1 Processing is Associated with Synaptic and Behavioral Abnormalities in a Prenatal Valproic Acid Model of Autism

Yu-Jin Kim[1], Han-Byeol Kim[1], Hyo-Min Lim[1], Yoori Choi[2], Ran-Sook Woo[1*]

[1] Department of Anatomy and Neuroscience, College of Medicine, Eulji University, Daejeon, 34824, Republic of Korea

[2] Department of Nuclear Medicine, Seoul National University Hospital, Seoul, 03080, Republic of Korea,

* Correspondence to: Ran-Sook Woo

Department of Anatomy and Neuroscience, College of Medicine, Eulji University, 143-5, Yongdu-Dong, Jung-Gu, Daejeon, Republic of Korea

E-mail: rswoo@eulji.ac.kr

Supplementary Video 1. Impaired impulsive-like behavior in VPA male rats during the cliff avoidance test. In the cliff avoidance test, VPA male rats showed markedly reduced latency to fall from the platform. Systemic NRG1 administration significantly increased latency in VPA rats, indicating reduced impulsive-like behavior.

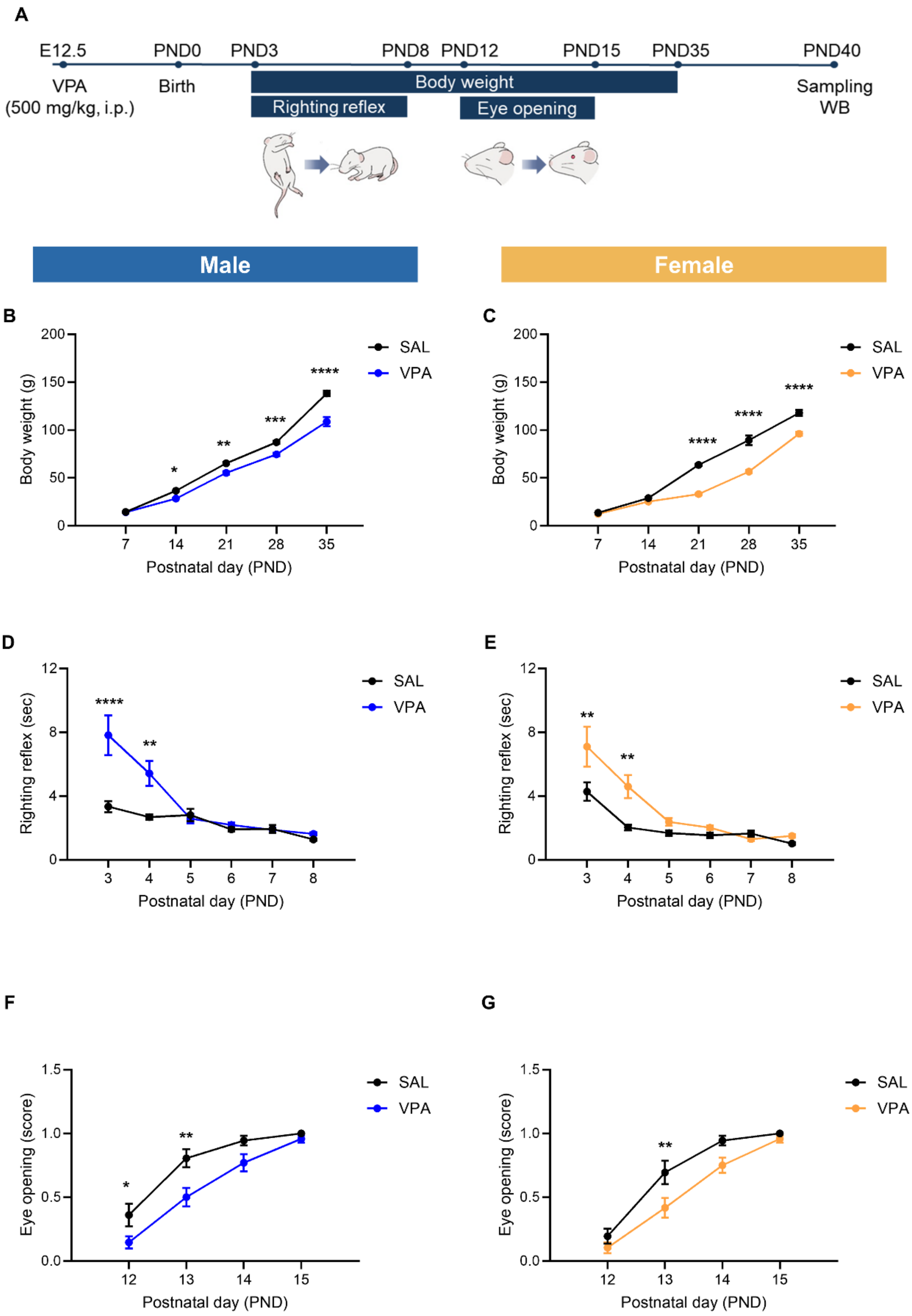
A
E12.5
PND0
PND3
PND8
PND12
PND15
PND35
PND40
VPA
(500 mg/kg, i.p.)
Birth
Body weight
Righting reflex
Eye opening
Sampling
WB
Male
Female
B
C
Body weight (g)
Postnatal day (PND)
SAL
VPA
D
E
Righting reflex (sec)
F
G
Eye opening (score)

Supplementary Figure 1. Early developmental trajectories in the valproic acid (VPA) rat model. (**A**) Experimental timeline showing maternal VPA (500 mg kg^{-1}, i.p.) at E12.5 and postnatal assessments (body weight PND7–35, righting reflex PND3–8, eye opening PND12–15) with adolescent tissue sampling. (**B**) Male body weight is reduced in the VPA model from PND14–35. n = 19 rats in SAL; n = 22 rats in VPA. (**C**) Female body weight shows a similar reduction across PND21–35. n = 18 rats in SAL; n = 24 rats in VPA. (**D**) Male righting reflex latency is prolonged at PND3–4 and converges by PND7–8. n = 18 rats in SAL; n = 21 rats in VPA. (**E**) Female righting reflex latency shows the same pattern. n = 18 rats in SAL; n = 24 rats in VPA. (**F**) Male eye opening score is delayed at PND12–13 and converges by PND15. n = 18 rats in SAL; n = 24 rats in VPA. (**G**) Female eye opening score shows the same delay and convergence. n = 18 rats in SAL; n = 24 rats in VPA. Data are mean ± SEM of biologically independent animals with individual values shown. Statistics: Two-way repeated-measures ANOVA (group × day) followed by Tukey's multiple-comparisons test (**B-G**). *$p<0.05$, **$p<0.01$, ***$p<0.001$, and ****$p<0.0001$.

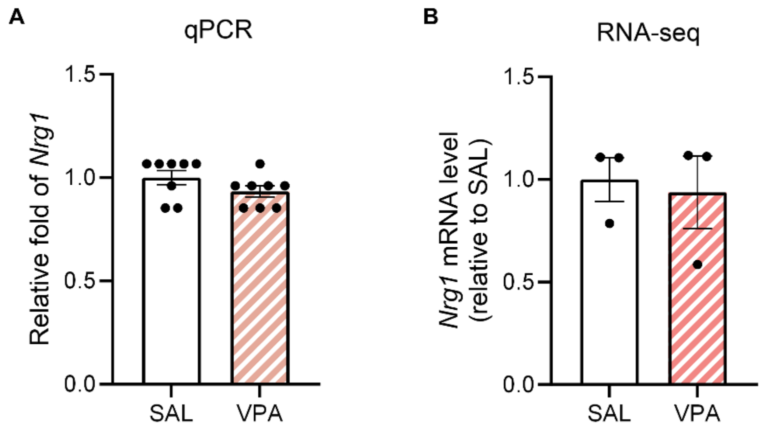

Supplementary Figure 2. NRG1 mRNA expression is unchanged in the VPA model.

(**A**) Relative expression of *Nrg1* mRNA measured by quantitative PCR (qPCR), normalized to the reference gene and scaled to the SAL group. (**B**) Transcriptomic analysis using QuantSeq 3′ mRNA sequencing shows no significant change in *Nrg1* mRNA levels between SAL and VPA groups. Data are mean ± SEM of biologically independent animals with individual values shown. No significant differences were detected. Sample sizes: qPCR, $n = 8$ per group; RNA-seq, $n = 3$ per group.

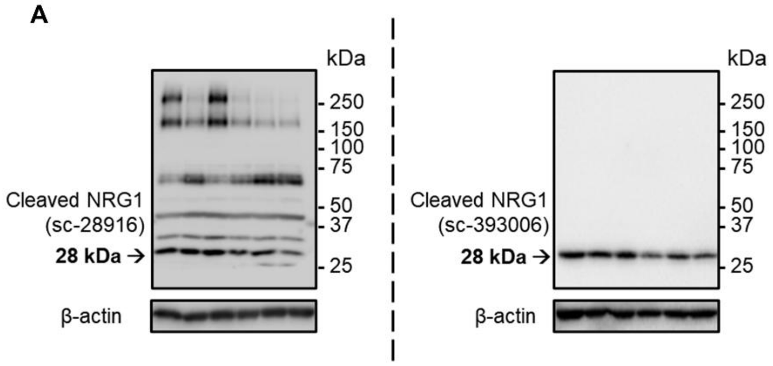

Supplementary Figure 3. Full-length uncropped immunoblots for cleaved NRG1.

Full-length, uncropped immunoblot images corresponding to Fig. 1B are shown. Cleaved NRG1 was detected using two independent antibodies (sc-28916 and sc-393006), with β-actin used as a loading control. Molecular weight markers (kDa) are indicated (~ 28 kDa). These full blot images are provided to allow independent assessment of band specificity and signal integrity.

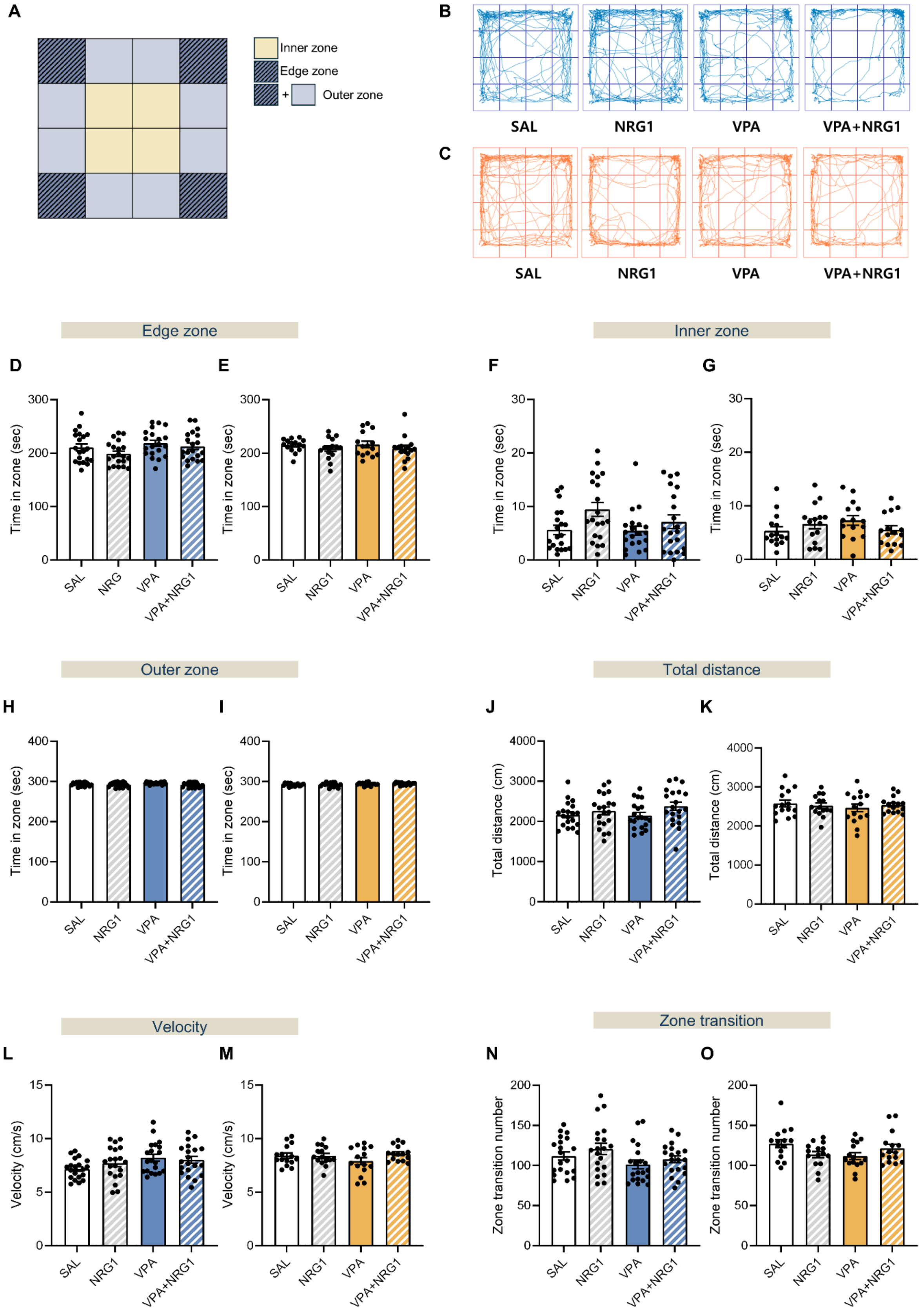
A
Inner zone
Edge zone
Outer zone
B
SAL
NRG1
VPA
VPA+NRG1
C
SAL
NRG1
VPA
VPA+NRG1
Edge zone
Inner zone
D
E
F
G
Time in zone (sec)
SAL
NRG
NRG1
VPA
VPA+NRG1
Outer zone
Total distance
H
I
J
K
Total distance (cm)
Velocity
Zone transition
L
M
N
O
Velocity (cm/s)
Zone transition number

Supplementary Figure 4. Open-field locomotion is unchanged across groups. (**A**) Schematic of inner, edge, and outer zones. (**B**) Representative locomotor tracks (males). (**C**) Representative locomotor tracks (females). (**D**) Male time in edge zone shows no group difference. (**E**) Female time in edge zone shows no group difference. (**F**) Male time in inner zone shows no group difference. (**G**) Female time in inner zone shows no group difference. (**H**) Male time in outer zone shows no group difference. (**I**) Female time in outer zone shows no group difference. (**J**) Male total distance traveled shows no group difference. (**K**) Female total distance traveled shows no group difference. (**L**) Male velocity shows no group difference. (**M**) Female velocity shows no group difference. (**N**) Male zone transitions show no group difference. (**O**) Female zone transitions show no group difference. $n = 20$ per group in males; $n = 15$ per group in females. Data are means ± SEM with individual animals shown**.** Statistics: One-way ANOVA followed by Tukey's multiple-comparisons test within sex (**D-O**).

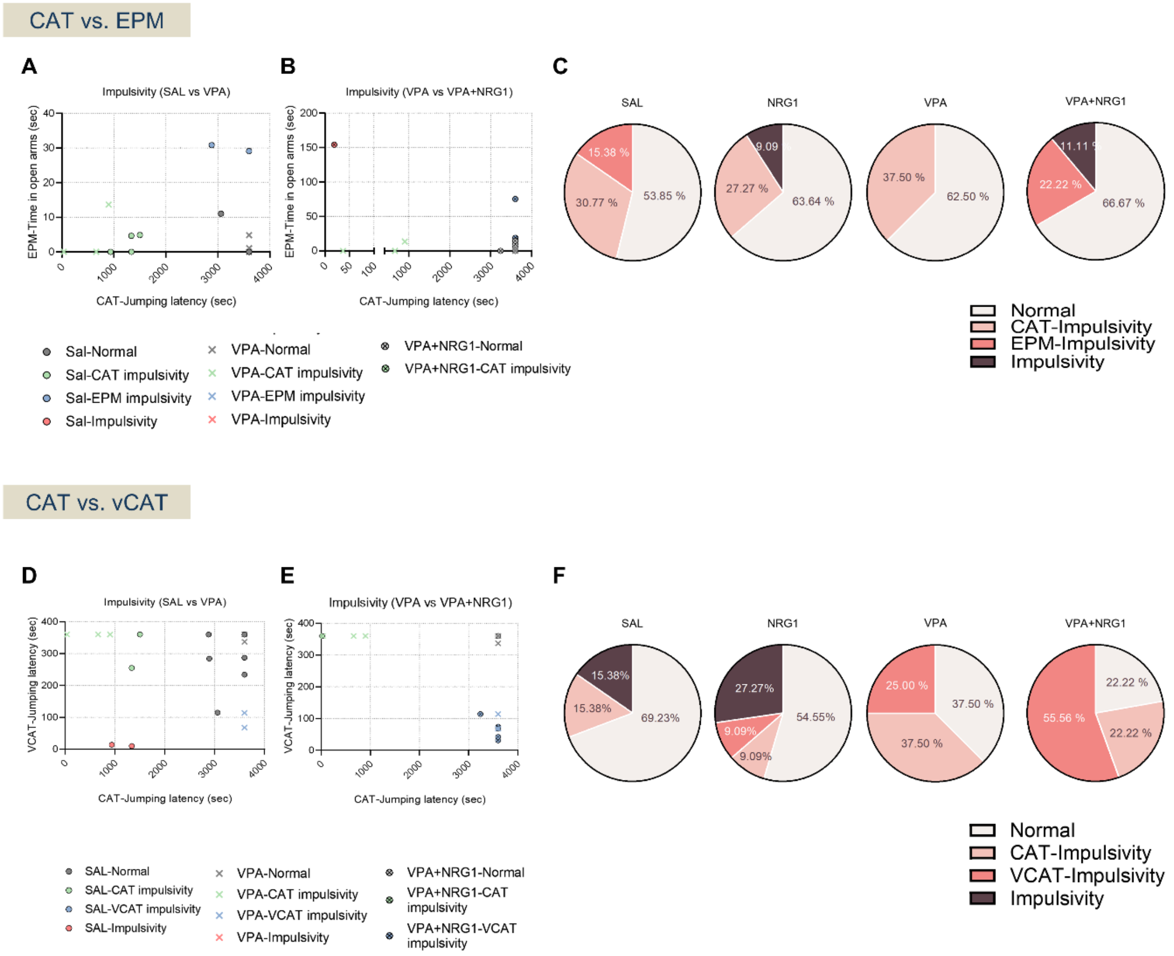

Supplementary Figure 5. Cross-assay integration in the VPA female rat model. (**A**) Female scatter of CAT jumping latency versus EPM open-arm time for SAL versus VPA. (**B**) Female scatter of CAT jumping latency versus EPM open-arm time for VPA versus VPA+NRG1. (**C**) Female pie charts showing category proportions across groups. (**D**) Female scatter of CAT jumping latency versus vCAT jumping latency for SAL versus VPA. (**E**) Female scatter of CAT jumping latency versus vCAT jumping latency for VPA versus VPA+NRG1. (**F**) Female pie charts showing category proportions for the CAT–vCAT pairing. Classification rules for categories were predefined in the Methods section; each symbol represents one animal. Statistics: Fisher’s exact test with Holm–Bonferroni correction for differences in category proportions.

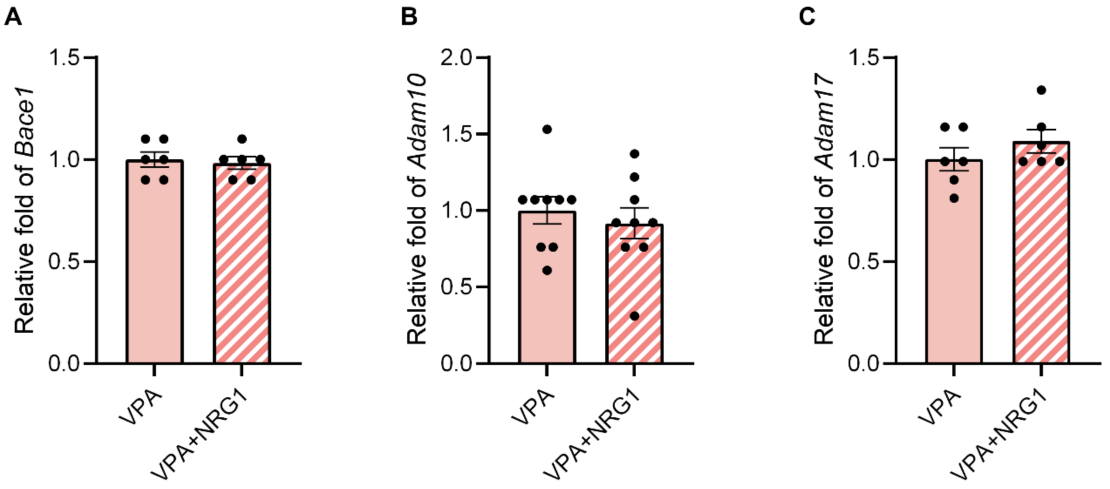

Supplementary Figure 6. Sheddases transcripts are unchanged by NRG1 in male hippocampus. (**A**) *Bace1* mRNA (qPCR) shows no difference. n = 6 per group. (**B**) *Adam10* mRNA shows no difference between VPA and VPA+NRG1. n = 9 per group. (**C**) *Adam17* mRNA shows no difference. n = 6 per group. Bar graphs show means ± SEM with individual animals; values are normalized to reference genes and scaled to the VPA mean. Statistics: Two-sided Student's *t*-tests (**A-C**).
